# The potential role of vitamin D supplementation as a gut microbiota modifier in healthy individuals

**DOI:** 10.1038/s41598-020-77806-4

**Published:** 2020-12-10

**Authors:** Parul Singh, Arun Rawat, Mariam Alwakeel, Elham Sharif, Souhaila Al Khodor

**Affiliations:** 1 Research Department, Sidra Medicine, Doha, Qatar; 2 College of Health Sciences, Qatar University, Doha, Qatar

**Keywords:** Microbiome, Predictive markers, Calcium and vitamin D

## Abstract

Vitamin D deficiency affects approximately 80% of individuals in some countries and has been linked with gut dysbiosis and inflammation. While the benefits of vitamin D supplementation on the gut microbiota have been studied in patients with chronic diseases, its effects on the microbiota of otherwise healthy individuals is unclear. Moreover, whether effects on the microbiota can explain some of the marked inter-individual variation in responsiveness to vitamin D supplementation is unknown. Here, we administered vitamin D to 80 otherwise healthy vitamin D-deficient women, measuring serum 25(OH) D levels in blood and characterizing their gut microbiota pre- and post- supplementation using 16S rRNA gene sequencing. Vitamin D supplementation significantly increased gut microbial diversity. Specifically, the *Bacteroidetes* to *Firmicutes* ratio increased, along with the abundance of the health-promoting probiotic taxa *Akkermansia* and *Bifidobacterium.* Significant variations in the two-dominant genera, *Bacteroides* and *Prevotella*, indicated a variation in enterotypes following supplementation. Comparing supplementation responders and non-responders we found more pronounced changes in abundance of major phyla in responders, and a significant decrease in *Bacteroides acidifaciens* in non-responders. Altogether, our study highlights the positive impact of vitamin D supplementation on the gut microbiota and the potential for the microbial gut signature to affect vitamin D response.

## Introduction

Vitamin D is a lipid-soluble vitamin that is absorbed from dietary sources or supplements in the proximal small intestine^1^, and is essential for maintaining skeletal integrity and function^2^, as well as for electrolyte reabsorption^3^, and immune system regulation^4^. In some populations, sub-clinical vitamin D deficiency is common, affecting close to 40% of individuals in both the US^5^ and Europe^6^, as well as 80–85% of people living in Arab countries^7–10^. This is of particular concern given recent studies revealing the association between vitamin D deficiency and a multitude of diseases including cancer, cardiovascular diseases^11–13^, diabetes, obesity^14,15^ and inflammatory bowel disease (IBD)^16,17^. In diabetes^18^ and IBD^19^, vitamin D is intimately involved in the regulation of inflammation via a bidirectional relationship with the gut microbiota^20,21^. Studies also suggest that the amount of dietary vitamin D and its circulating levels may be involved in maintaining immune homeostasis in healthy individuals, partially via modulating the gut microbial composition^22^. However, it is currently unknown how supplementing otherwise-healthy vitamin D-deficient people affects their gut microbiota.

Several studies have assessed the impact of vitamin D supplementation on the microbiota composition, predominantly in disease states. For example, Kanhere et al. showed that weekly vitamin D supplementation modifies the gut and airway microbiota in patients with cystic fibrosis^23^. In another study, vitamin D3 supplementation of patients with multiple sclerosis increased abundance of the mucosal-integrity-promoting genus *Akkermansia* in the gut, as well as *Fecalibacterium* and *Coprococcus*; these latter two being the major butyrate producers of the Firmicutes phylum^24^. Similarly, in vitamin D-deficient pre-diabetic individuals, supplementation leading to increased serum 25(OH) D was inversely correlated with abundance of *Firmicutes* (genus *Ruminococcus*) and *Proteobacteria*, and positively correlated with *Bacteroidetes* abundance^22,25,26^. A randomized clinical trial in vitamin D-deficient overweight or obese adults also showed that increased levels of vitamin D were associated with greater abundance of bacteria from the genus *Coprococcus* and lower abundance of the genus *Ruminococcus*^27^.

Studies examining the effect of vitamin D supplementation on the gut microbiota composition of healthy individuals are limited. In one study, increased relative abundance of *Bacteroidetes* and decreased *Proteobacteria* was reported, but only in biopsies from the upper gastrointestinal tract and not in fecal samples^28^. However, a small study with twenty healthy Vitamin D-deficient/insufficient subjects showed a significant dose-dependent increase in the relative abundance of *Bacteroides* and *Akkermansia spp,* coupled with a decrease in Firmicutes-to-Bacteroidetes ratio and decreased relative abundance of *Fecalibacterium *spp. and the *Ruminococcaceae* family^29^. Thus, there is some controversy around the effects of vitamin D supplementation of healthy individuals on the gut microbiota and whether these effects are significant in the lower gastrointestinal tract of a large study population.

Further complicating our understanding of the impact of vitamin D deficiency and the effects of supplementation is the observation that changes in serum levels of the vitamin D pre-hormone metabolite, 25(OH)D (25-hydroxyvitamin D), post-supplementation vary widely among individuals^30–33^, with around 25% of people demonstrating little or no increase in blood 25(OH)D following vitamin D_2_/D_3_ supplementation^34^. A systematic review by Zittermann et al. concluded that individual variations in serum 25(OH) D levels post supplementation could be partly explained by differences in dose per kg of body weight (34.5%), the type of supplement used (D_2_ or D_3_, 9.8%), age (3.7%), concurrent calcium supplementation (2.4%) and baseline 25(OH)D concentration (1.9%)^35^; however, this leaves 50% of the inter-individual difference in response unaccounted for. Given the evidenced bi-directional interaction between vitamin D and the gut microbiota in inflammation, we hypothesized that the composition of the gut microbiota might also affect responsiveness to vitamin D intake.

Therefore, in this study we characterized the composition and diversity of the gut microbiota in a group of healthy adult females before and after supplementation with vitamin D, and established both the effects of supplementation on gut microbiota and whether specific microbial signatures were associated with the differential serum response to oral vitamin D supplements.

## Results

### Participant characteristics and the effects of vitamin D supplementation on blood biochemistry

**Figure 1:**
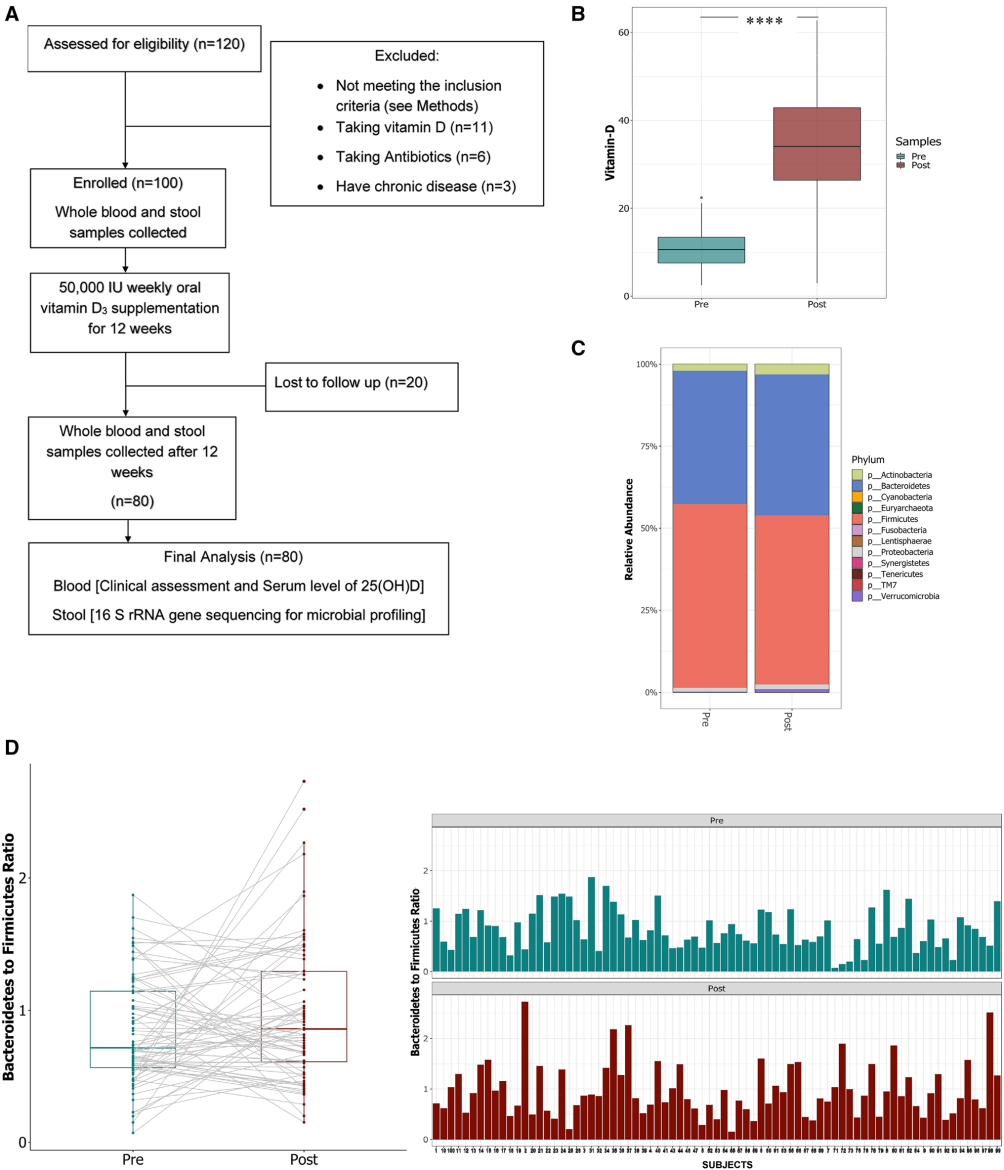
The schematic representation of study design and analysis. (**A**) Flow chart of subject selection along with inclusion/exclusion criteria. (**B**) Changes in serum levels of vitamin D(ng/ml) in study subjects pre- and post- supplementation. Microbiota composition in stool samples pre- and post- vitamin D supplementation. (**C**) The relative abundance of bacterial phyla: *Firmicutes* and *Bacteroidetes* were significantly impacted post Vitamin D (Wilcoxon test with false discovery rate (FDR)-Bonferroni corrected ****P < 0.0001 and *P < 0.05 respectively) (**D**) Comparison of the ratio of *Bacteroidetes* to *Firmicutes* pre- and post- vitamin D supplementation (Lmer4 borderline significant *p* = 0.0579) cumulative and per subject level. The figure was generated using (RStudio v 1.2 with R v 3.6)^87^.

We enrolled 100 healthy female subjects into the study, of which 80 successfully completed the two phases (phase I-baseline-pre-supplementation; phase II- post-supplementation with vitamin D3). The study workflow and exclusion criteria are shown in (Fig. 1A). Briefly, following enrollment, blood and stool samples were collected; all participants were then given a weekly oral dose of 50,000 IU vitamin D_3_ to be taken for the following 12 weeks, at which time a second set of blood and stool samples were taken, the phase I and phase II samples were analyzed for serum 25(OH) and gut microbiota composition. Baseline clinical and demographic characteristics of the participants are summarized in (Table 1). Briefly, the mean age of the cohort was 21 years, and 87% of the participants were Arabs. The average body mass index (BMI) of the subjects was 24.39 ± 0.530 kg/m^2^, with the majority of individuals falling into the normal weight category.

**Table 1:** Baseline Characteristics of Study Participants.

Characteristic	Measure	
**Age, mean** (range in years)	21(17–28)	
**Ethnicity, n (%)**		
Arab	70 (87.5%)	
Non- Arab	10 (12.5%)	
**BMI, mean ± SEM**	24.39 ± 0.530	
**Classification according to BMI**		
Underweight, n (%)	4 (5%)	
Normal weight, n (%)	52 (65%)	
Overweight, n (%)	14 (17.5%)	
Obese, n (%)	10 (12.5%)	
**Average Daily Exposure to Sun**		
Less than 1/2 h, n (%)	30 (37.5%)	
1/2 h to 1 hr, n (%)	32 (40%)	
more than 1 h, n (%)	18 (22.5)	
**Frequency of fish consumption**		
Daily, n (%)	0 (0%)	
Weekly, n (%)	16 (20%)	
Monthly, n (%)	40 (50%)	
None, n (%)	24 (30%)	
**History of Vitamin D deficiency**		
Yes (%)	76%	
No (%)	24%	
**Biochemical parameters**	Pre	Post
Serum 25(OH)D level, ng/ml, means ± SEM	11.03 ± 0.521	34.37 ± 1.476
Calcium(mg/dl), means ± SEM	9.18 ± 0.146	11.34 ± 0.165
Creatinine(mg/dl), means ± SEM	0.46 ± 0.013	0.677 ± 0.017
BUN (mg/dl), means ± SEM	9.81 ± 0.318	12.61 ± 0.393
ALT(U/L), means ± SEM	9.86 ± 0.521	13.01 ± 0.721
AST(U/L), means ± SEM	15.14 ± 0.558	16.46 ± 0.613

SEM, Standard error of measurement; BMI, Body mass index; BUN, blood urea nitrogen; ALT, alanine aminotransferase; AST, aspartate aminotransferase.

At the start of the study, participants had 25(OH)D levels classed as either deficient (less than 20 ng/ml, 96% of all participants) or insufficient (less than 30 ng/ml, 4% of the remaining participants) according to published limits^36^. This is consistent with the most recent Qatar Biobank report showing over 88% of the population has inadequate levels of vitamin D^10^. After 12 weeks of vitamin D supplementation in the absence of significant self-reported dietary change, we found that average serum 25(OH) D levels had increased significantly across the group (baseline 11.03 ± 0.51 ng/ml to post-supplementation 34.37 ± 1.47 ng/ml (*p* = 5.1e−14; paired Wilcoxon, Fig. 1B). Overall, 89% of participants achieved a serum level of 25(OH)D > 20 ng/ml, with 69% reaching a sufficient level exceeding 30 ng/ml (data not shown). The 11% of subjects that remained deficient (< 20 ng/ml 25(OH)D) in vitamin D despite supplementation were classified as non-responders^37,38^. As expected, we also found that average calcium concentration increased significantly post-vitamin D supplementation (Table 1 and Supplementary Fig. S1A).

As vitamin D deficiency is associated with chronic liver^39^ and kidney^40^ diseases, we also measured markers of the function of these organs. We found that vitamin D supplementation significantly decreased the ratio of serum blood-urea-nitrogen (BUN)/Creatinine, indicating improved kidney function, as well as decreasing the ratio of aspartate aminotransferase (AST)/ alanine aminotransferase (ALT), indicative of improved liver functioning (Table 1 and Supplementary Fig. S1B/C). These results are consistent with a study showing that kidney function (BUN/Creatinine ratio) improved in vitamin D-deficient patients who took vitamin D supplements than those that didn’t^41^. Similarly, a cross sectional study of 5528 school students found that abnormal liver function tests were corrected (the AST/ALT ratio was decreased) post vitamin D supplementation^42^. Taken together, we show that weekly oral supplementation of vitamin D in healthy females was effective in restoring healthy levels of blood 25(OH)D in majority of the participants. Moreover, this increase was associated with increased blood calcium levels and improvements to blood markers of kidney and liver function in this cohort.

### Effects of vitamin D supplementation on gut microbiota composition

We next determined the bacterial composition of stool samples from participants before and after 12 weeks of vitamin D supplementation using 16S rRNA gene sequencing on the Illumina MiSeq platform. We generated 9.4 million (9,405,441) paired-end sequences of the 16S rRNA genes from the 80 subjects providing samples pre- and post- supplementation. The mean number of sequences was 58,784 ± 31,109 per sample. After de-noising, we defined 7,332 operational taxonomic units (OTUs), with a mean length of 411.5 ± 19.19 bp. These OTUs were classified into 12 different phyla, as shown in the prevalence plot in (Supplementary Fig. S2).

The adult human gut is generally predominantly populated by bacteria within the phyla *Bacteroidetes* and *Firmicutes*^43^; as well as the less abundant *Actinobacteria*, *Proteobacteria*, and *Verrucomicrobia*^44^. Accordingly, here we found that, pre-supplementation, *Firmicutes* and *Bacteroidetes* represented around 95% of the total sequencing reads: the mean relative abundance of *Firmicutes* (55.86%) and *Bacteroidetes* (40.70%) were by far the highest across all the samples we analyzed, followed by *Actinobacteria* (2.00%), *Proteobacteria* (1.15%) and *Verrucomicrobia* (0.21%) (Fig. 1C). However, following vitamin D supplementation, the mean relative abundance of *Firmicutes* decreased significantly to 50.57% (*p* < 2.2e−16), while the mean relative abundance of *Bacteroidetes* increased significantly to 43.62% (*p* = *0.001*) (Fig. 1C). Using a mixed model with repeated measures (lme4)^45^ we confirmed that the 12 week supplementation with vitamin D impacted the *Bacteroidetes*/*Firmicutes* (B/F) ratio. Our data showed that the B/F ratio was higher post vitamin D supplementation (0.818 ± 0.048 vs. 0.954 ± 0.061; *p* = 0.0579) (Fig. 1D). Among other phyla, the relative abundance of *Actinobacteria* (pre-1.9% vs post- 3.1%) and *Verrucomicrobia* (pre-0.19% vs post-0.95%) also increased (Fig. 1C).

**Figure 2:**
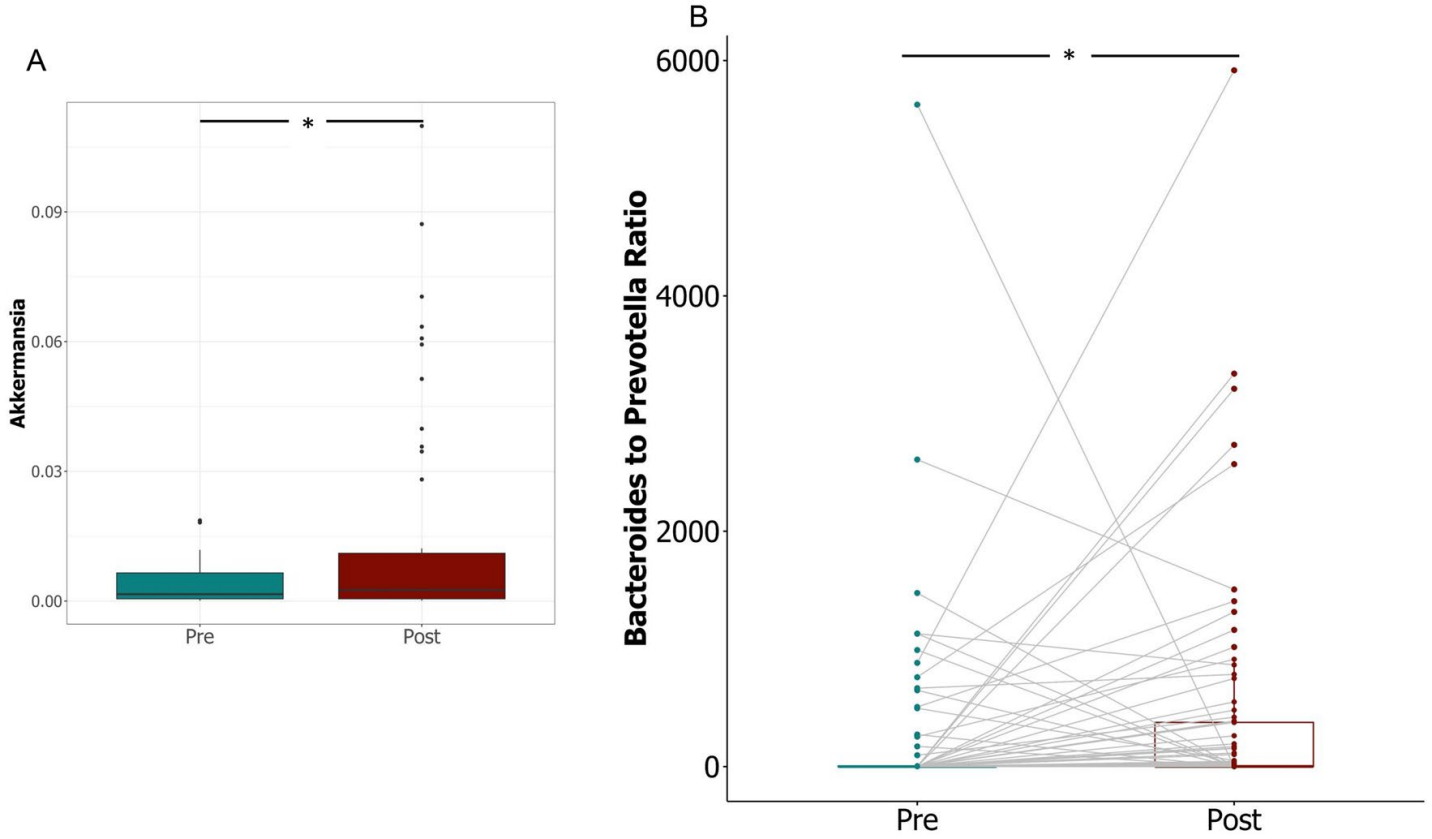
Changes in relative abundance of specific bacterial genera in stool samples pre- and post- vitamin D supplementation. (**A**) Relative abundance of genus *Akkermansia* (Wilcoxon test with false discovery rate (FDR)-corrected pairwise *P* values. *P < 0.05) (**B**) Comparison of the ratio of *Bacteroides* to *Prevotella* pre- and post- supplementation; (Wilcoxon test with false discovery rate (FDR)-Bonferroni corrected pairwise *P* values. *P < 0.05) The figure was generated using (RStudio v 1.2 with R v 3.6)^87^.

At the genus level, pair-wise comparison of the top 10 most abundant genera from each phylum revealed significant increases in the relative abundance of *Bifidobacterium* (predominant genus in *Actinobacteria*) and *Akkermansia* (only known member of phylum *Verrucomicrobia*) following vitamin D supplementation (*p* = 0.018) (Fig. 2A, Supplementary Fig. S3). In contrast, the abundance of several core genera in the phylum *Firmicutes*, such as *Roseburia*, *Ruminococcus*, and *Fecalibacterium* decreased post supplementation (Supplementary Figs. S4 and S6); whereas members of the phylum *Bacteroidetes* showed an increase in relative abundance of the genera *Bacteroides, Alistipes* and *Parabacteroides,* and a decrease in *Prevotella* (Supplementary Figs. S5 and S6). The change in the relative abundance of the two dominant genera within *Bacteroidetes*, *Bacteroides* and *Prevotella* (marked by a significant increase in the *Bacteroides/Prevotella* ratio, *p* = 0.0057) (Fig. 2B) combined with the decreased abundance of *Ruminoccoccus* indicates a shift of enterotypes in favour of the *Bacteroides*-dominated enterotype (ET B)^43^*.* Altogether the results indicate that vitamin D supplementation results in alteration of the composition of both the major and minor phyla in the gut of healthy individuals.

### Effects of vitamin D supplementation on richness and diversity of the gut microbiota

**Figure 3:**
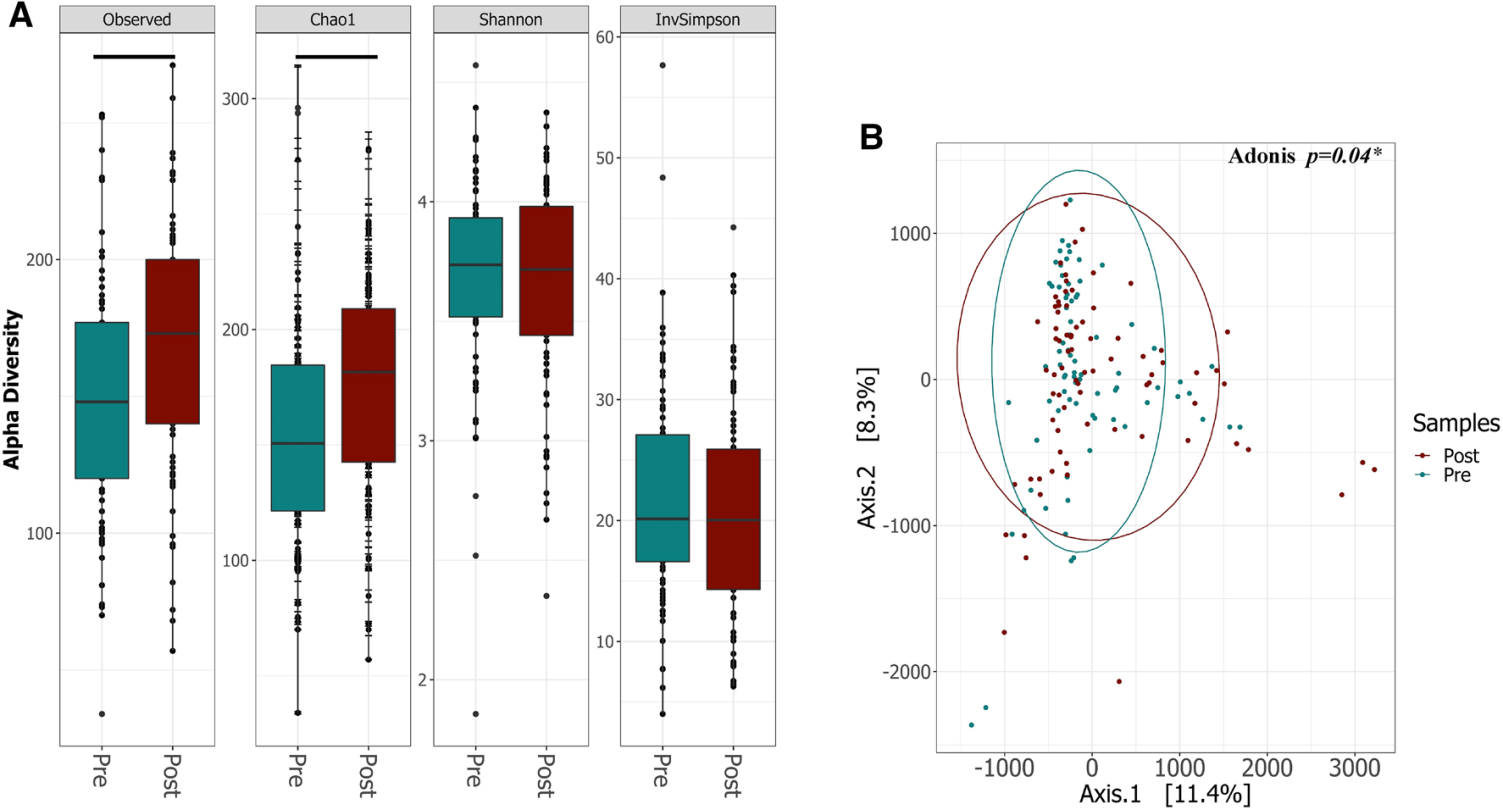
Diversity of microbiota composition in stool samples pre- and post- vitamin D supplementation. (**A**) Boxplots of Alpha-diversity indices: Observed OTUs; Chao1; Shannon and Inverse Simpson. Boxes represent the interquartile range (IQR) between the first and third quartiles (25th and 75th percentiles, respectively), and the horizontal line inside the box defines the median. Whiskers represent the lowest and highest values within 1.5 times the IQR from the first and third quartiles, respectively. Statistical significance was identified by the Wilcoxon test with false discovery rate (FDR)-Bonferroni corrected pairwise *P* values. *P < 0.05; **P < 0.01; ***P < 0.001 and ****P < 0.0001. (**B**) PCA on a weighted UniFrac dissimilarity matrix shows significant differences in β diversity of bacterial populations pre- and post- vitamin D supplementation, with higher variance post supplementation. *P < *0.05)* The figure was generated using (RStudio v 1.2 with R v 3.6)^87^.

In contrast to previous studies, we found a significant impact of vitamin D supplementation on both alpha and beta diversity of the gut microbiota in healthy females. At the end of the 12 week supplementation period, we observed a statistically significant increase in the observed OTUs *(p* = 1.6e−05) and Chao1 indices (*p* = 1.1e−05), whereas the Shannon and InvSimpson indices were not significantly different (*p* = *0.71* and *p* = 0.27 respectively) (Fig. 3A). When we evaluated the overall structure of the fecal microbiota using β diversity indices, we found a significant difference in the weighted UniFrac dissimilarity matrix between the two groups (PERMANOVA *p* = *0.048*) (Fig. 3B).

Thus, the above results suggest the diversification of gut microbiota in healthy adult females post vitamin D supplementation.

### Association of microbial signatures with response to vitamin D supplementation

**Figure 4:**
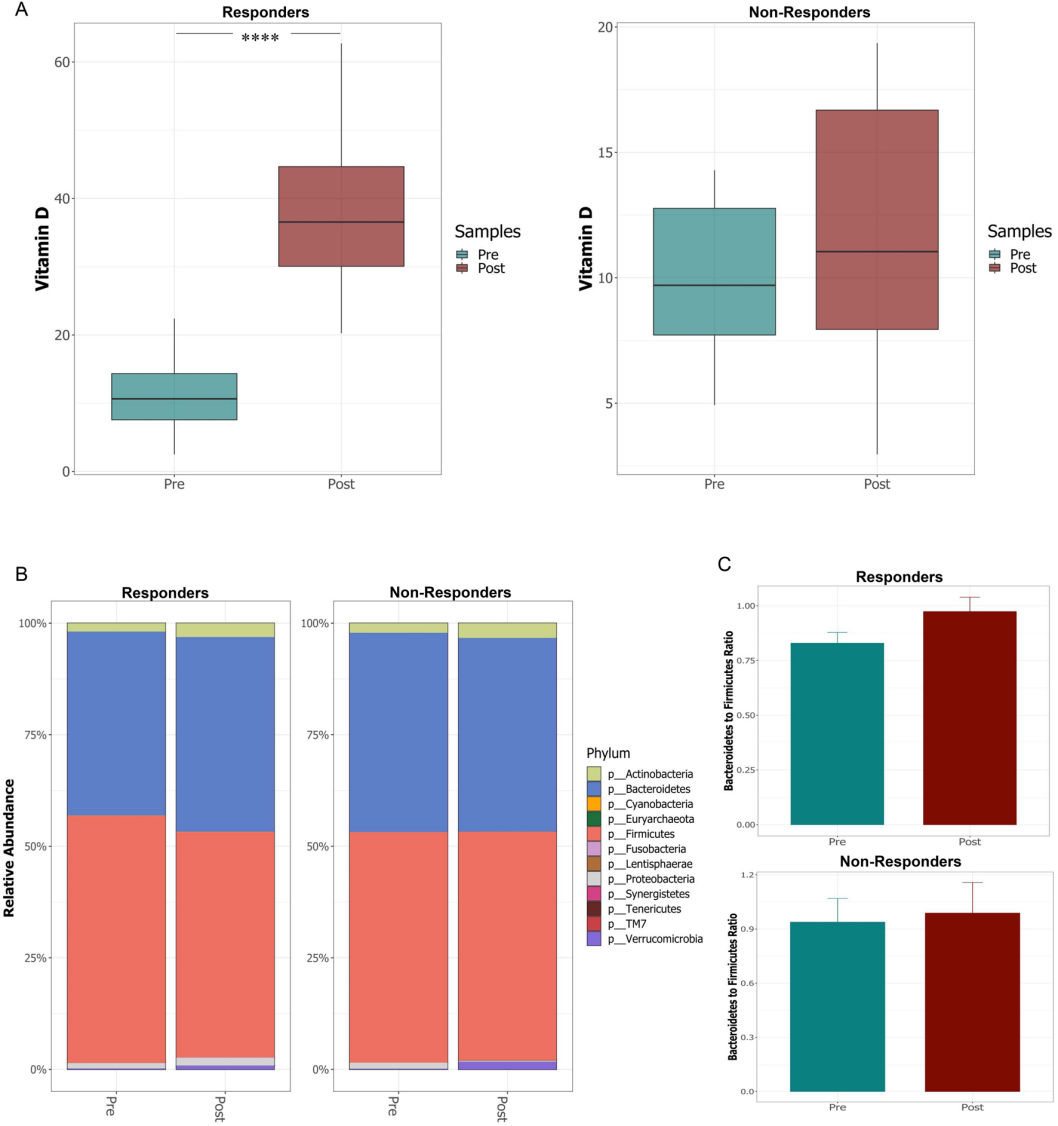
Comparison of changes in serum vitamin D levels (ng/ml) and gut microbiota composition in responders and non-responders to vitamin D supplementation. (**A**) Serum vitamin D levels pre- and post- supplementation in responders and non-responders. (**B**) Relative abundance of different bacterial phyla pre and post supplementation in responder and non-responder groups. (**C**) The ratio of *Bacteroidetes* to *Firmicutes* in responders and non-responders, pre- and post- vitamin D supplementation. The figure were generated using (RStudio v 1.2 with R v 3.6)^87^.

Studies show a high interpersonal variability in the response to vitamin D supplementation, the reasons for which are incompletely understood. Given the bi-directional relationship between vitamin D and the microbiota in inflammation, we hypothesized that a similar interaction might occur in determining responsiveness to vitamin D supplementation. We thus categorized our subjects as responders or non-responders based on their vitamin D levels post supplementation: responders were defined as those who achieved serum levels of 25(OH) D above 20 ng/ml and the non-responders were those whose serum levels of 25(OH) D remained < 20 ng/ml) (Fig. 4A)^37,38^.

**Figure 5:**
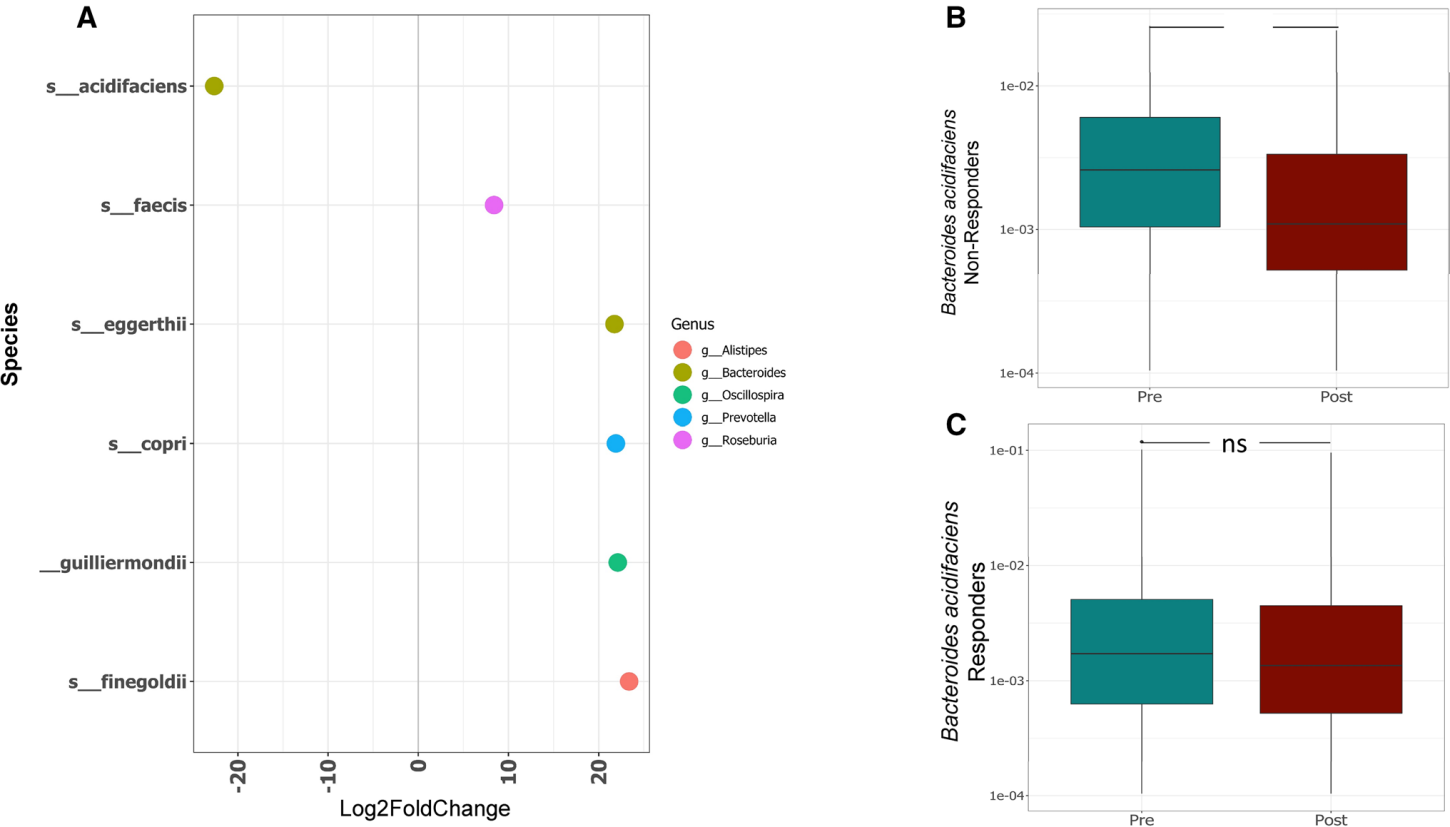
Species level comparison within gut microbiota of responders and non-responders to vitamin D supplementation. (**A**) DESeq2 differential abundance analysis of significantly different OTUs post/pre in non-responders (*p* < 0.05, FDR-corrected); OTUs to the right of the zero line were more abundant in non-responders post- supplementation and OTUs to the left of the zero line were less abundant. (**B**–**C**) Comparison of relative abundance of *B. acidifaciens* in non-responders (**B**) and responders (**C**) pre- and post-vitamin D supplementation. Significant decrease in non-responders post supplementation (**P < 0.01). Responders show non-significant change. Statistical significance was identified by the Wilcoxon test with false discovery rate (FDR)-Bonferroni corrected pairwise *P* values. *P < 0.05; **P < 0.01). The figure was generated using (RStudio v 1.2 with R v 3.6)^87^.

We next asked whether the two groups differed with respect to changes in gut microbial composition during supplementation. The two groups ordinated based on their treatment status (pre/post supplementation; PERMANOVA *p* = 0.048) (Supplementary Fig. S7); as well as segregating into responders and non-responders, based on the variation in the microbiota composition as a result of vitamin D supplementation. Vitamin D responders showed significant increases in the relative abundance of *Bacteroidetes(p* = 0.012), Actinobacteria (*p* = 0.010), Proteobacteria (*p* = 0.005) and Lentisphaeraea (*p* = 0.05), coupled with decreased abundance of Firmicutes (*p*  < 2.2e−16) at the phylum level post supplementation (Fig. 4B); In non-responders changes were observed in the abundance of *Proteobacteria(p* = *0.02)*. Vitamin D responders also showed a greater increase in the B/F ratio post-supplementation, compared to non-responders (Fig. 4B). At the species level, we performed a differential abundance analysis using DESeq2 to compare responders and non-responders pre and post-vitamin D supplementation. Several microbes including *Bacteroides acidifaciens, Ruminococcus bromii*, *Bacteroides eggerthii*, *Barnesiella intestinihominis* were found to be significantly enriched in responders compared to non-responders both in pre and post-supplementation *(padj* < *0.05)* (Supplementary Fig. S8A/B), suggesting that the enrichment with these microbes may be associated with the response to vitamin D supplementation. We next asked the question, which among these species were further depleted specifically in non-responders post-supplementation. Our analysis revealed a significant depletion of *B. acidifaciens* compared to other species in non-responders post supplementation (*padj* < 0.05) (Fig. 5A), which was also confirmed by Wilcoxon paired test (Fig. 5B/C). These results suggest that lower baseline levels of *B. acidifaciens* prior to vitamin D supplementation, combined with its continued depletion post supplementation may be indicative of poor response to vitamin D.

Both responders and non-responders showed an increase in alpha diversity post supplementation, as per the Observed and Chao 1 indices (data not shown). Collectively the signatures revealed that vitamin D supplementation has a differential modulatory effect on the microbial composition of the gut in responders and non-responders. While both groups exhibit changes in microbial composition and diversity following supplementation, the specifics of this change vary dependent on response status.

### Predicted functional profiling of the gut microbial communities pre- and post- vitamin D supplementation

**Figure 6:**
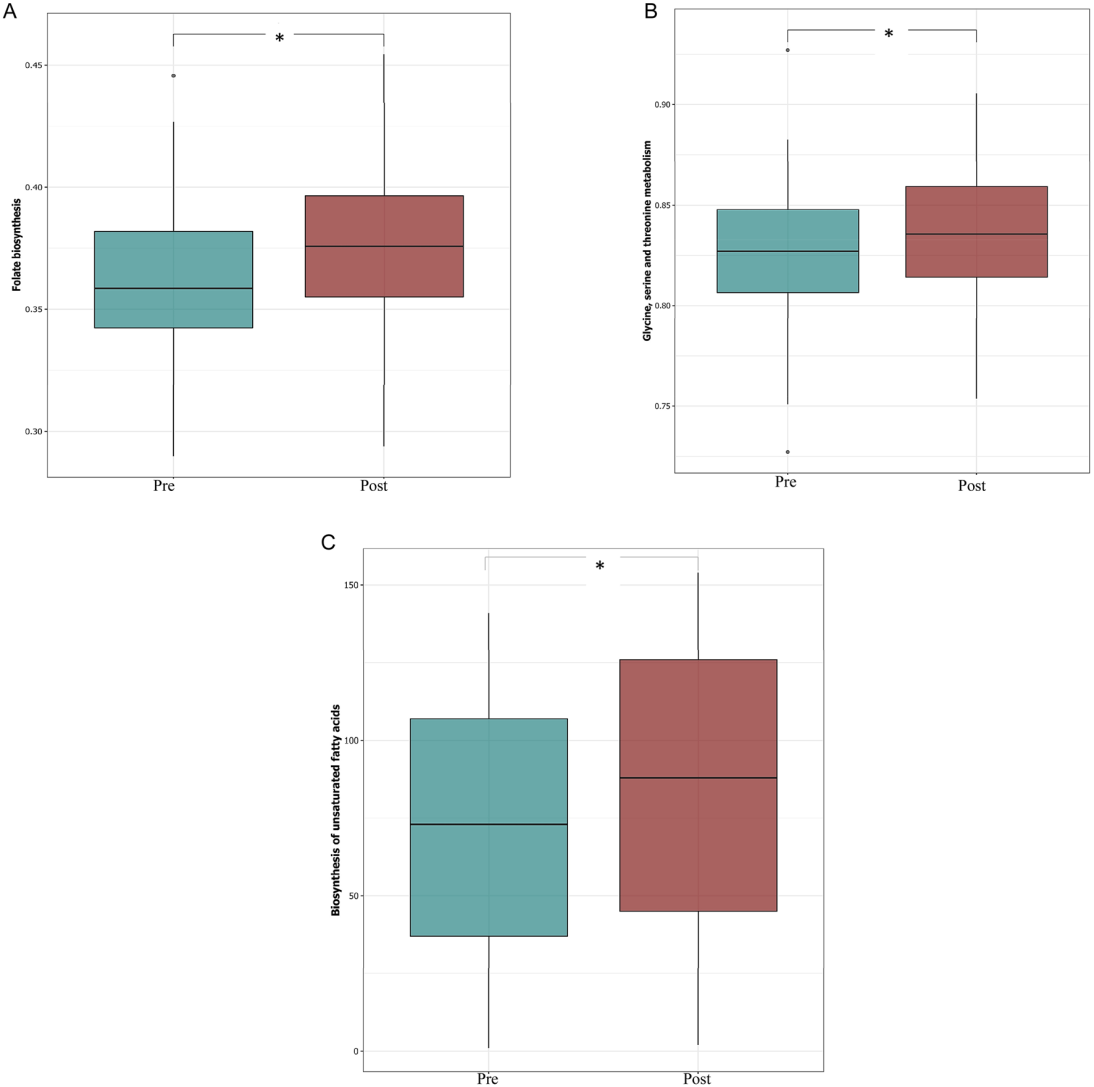
Inferred gut microbiome functions by PICRUSt from 16S rRNA gene sequences pre- and post- vitamin D supplementation. Difference in predicted functions of genes involved in (**A**) biosynthesis of folate; and (**B**) glycine, serine and threonine metabolism (**C**) biosynthesis of unsaturated fatty acids (Mann–Whitney*P < 0.05;). The figure was generated using (RStudio v 1.2 with R v 3.6)^87^.

To predict the functional role of the microbial communities identified, we used PICRUSt analysis^46^. Our data revealed marked differences between predicted patterns of functional genes pre- and post- vitamin D supplementation (Supplementary Fig. S9). Importantly, we saw significant differences in genes related to host-symbiont metabolic pathways, including folate biosynthesis, and glycine, serine and threonine metabolism pre- and post- supplementation (Fig. 6A/B). Several strains of *Bifidobacterium* are able to produce folate^47,48^, thus this increase in the abundance of this genus may explain the predicted increase of folate biosynthesis. Moreover, the predicted increase in the bacterial glycine metabolism genes is potentially important, as lower plasma levels of glycine have been linked with obesity and type 2 diabetes^49^; bacterial glycine metabolism can vary with changes in microbiota composition and richness^50^, as seen in this study. Our analysis also predicted an increased in genes related to several pathways involved in lipid metabolism, fatty acid biosynthesis and metabolism of cofactors and vitamins post-vitamin D supplementation(Fig. 6C); this is particularly interesting because of the vital role of lipids and fatty acids in the absorption of vitamin D (fat soluble) in the intestinal lumen.

## Discussion

In this study we aimed to characterize changes in the gut microbiota of vitamin D-deficient female volunteers following 12 weeks of vitamin D supplementation. In addition, we wanted to assess whether any characteristics of the gut microbiota were linked with the response to vitamin D supplementation. We found that vitamin D supplementation increased the overall diversity of the gut microbiota, and in particular the increased the relative abundance of *Bacteroidetes* and decreased the relative abundance of *Firmicutes.* A high ratio of *Firmicutes* to *Bacteroidetes* has been correlated with obesity^51^ and other diseases^52–54^; while conversely a prebiotic intervention that decreased the *Firmicutes* to *Bacteroidetes* ratio resulted in improvements to gut permeability, metabolic endotoxemia and inflammation^55^. Alongside the results of a recent pilot study^29^, our data solidify the proposed link between Vitamin D supplementation and decreased *Firmicutes* to *Bacteroidetes* ratio, which is associated with improved gut health^54^.

In addition to improving the *Bacteroidetes* to *Firmicutes* ratio, our data show that members of *Verrucomicrobia* and *Actinobacteria phyla* also increased in abundance following vitamin D supplementation. *Akkermansia muciniphila* is the only representative of the phylum *Verrucomicrobia* in the human gut,^56,57^ and helps maintain host intestinal homeostasis by converting mucin into beneficial by‐products^58^. The abundance of *A. muciniphila* negatively correlates with body mass^59,60^ inflammation^61^ metabolic syndrome^62^ and both type 1 and type 2 diabetes^60,63^. Our analysis also showed a significant increase in the abundance of *Bifidobacterium* which is an important probiotic with a wide array of benefits to human health^64^, as well as playing a role in folate and amino acid production^65^. Accordingly, using PICRUSt predictive functional analysis, we predicted an increased in genes involved in folate production and biosynthesis of several amino acids following vitamin D supplementation.

Alongside characterising individual taxa, Wu et al. clustered fecal communities into two enterotypes distinguished primarily by the levels of *Bacteroides* and *Prevotella*, and found that vitamin D intake was negatively associated with abundance of the *Prevotella* enterotype, instead being strongly positively associated with the *Bacteroides* enterotype^66^. In line with this, we found that vitamin D supplementation favoured a *Bacteroide*s-dominated enterotype over *Prevotella.* This is potentially important as several studies indicate *Prevotella* as an intestinal pathobiont: high levels of *Prevotella* spp. have been reported in children diagnosed with irritable bowel syndrome^67^; while the expansion of *Prevotella copri* was strongly correlated with enhanced susceptibility to arthritis^68^.Taken together, our results make a compelling argument that vitamin D supplementation modulates the gut microbiota composition and diversity towards a more beneficial state—a previously undescribed benefit of vitamin D.

At present, the mechanism underlying vitamin D regulation of the gut microbiota is not clear. One possibility is that, following absorption in the small intestine^1^, vitamin D could impact gut microbial communities via indirect systemic mechanisms; for example, the vitamin D receptor (VDR) is highly expressed in the proximal colon and acts as a transcription factor regulating expression of over 1000 host genes involved in intestinal homeostasis and inflammation, tight junctions, pathogen invasion, commensal bacterial colonization, and mucosal defense^70^, including the defensins, cathelicidin, claudins, TLR2, zonulin occludens, and NOD2^69,70^. Interestingly, there is some recent evidence of the cross talk between the gut microbiota and VDR signalling affecting host responses and inflammation, and this appears to be bidirectional^9^. Intestinal VDR expression has been shown to regulates the host microbiota to mediates the beneficial effects of probiotics^71,72^ and vitamin D treatment^72–75^. Similarly, probiotics and pathogenic bacteria have been also shown to modulate VDR expression, with the former increasing^76^, and the latter decreasing^77^, its expression.

Alternatively, or alongside such systemic mechanisms, growing evidence suggests that vitamins administered in large doses escape complete absorption by the proximal intestine^78^, and so might then be available to directly modulate the distal gut microbiome. Whether this is the case for the vitamin D remains to be investigated; however, such a mechanism might account for the differences in microbiota change seen in various studies employing high versus low dose supplementation protocols.

Interestingly, in our study microbial functional potentials inferred using PICRUSt indicated that vitamin D supplementation elevated pathways associated with the metabolism of amino acids, cofactors, vitamins, and lipids, including steroid biosynthesis and fatty acid elongation. This could be important as adequate concentrations of lipids, bile salts and fatty acids are required for incorporation of fat-soluble vitamin D into mixed micelles, as a prerequisite for its absorption^79,80^. Thus, increased abundance of bacterial genes related to lipid and fatty acid metabolism post supplementation could indicate increased vitamin D bioavailability and absorption in the gut^81^.

While the benefits of vitamin D supplementation in deficient/insufficient level individuals are clear, there are a sub-group of people in which even high-dose oral vitamin D supplementation has been shown to be ineffective. A secondary aim of this study was to assess whether the microbiota in these individuals could be associated with their non-responder status. Lower levels of baseline *Bacteroides acidifaciens* in non-responders combined with an additional depletion post-supplementation suggest that this bacterium may be linked with response to vitamin D supplementation. *Bacteroides acidifaciens* has previously been proposed as a “lean bug” that could prevent obesity and improve insulin sensitivity^82^. It is also one of the predominant commensal bacteria that promote IgA antibody production in the large intestine. Thus, we hypothesize that the vitamin D supplementation promotes the ‘farming’ of good bacteria in order to maintain immune–microbe homeostasis.

While results from this study are promising and warrant more research, it is worth noting that our study has few limitations. Firstly, we did not have vitamin D sufficient controls to observe the impact of vitamin D supplementation in comparison with the deficient subjects. Secondly, addition of a placebo group would minimize the potential effects of non-treatment factors. Lastly, experimental studies with larger cohort needs to be undertaken to have sufficient representation of study responders/non-responders to confirm the finding of the present study.

In conclusion, vitamin D supplementation of deficient/insufficient otherwise healthy females changed the composition and diversity of the gut microbiota, eliciting a beneficial effect by improving health-promoting taxa along with clinical biomarkers for kidney and liver function. Our study also provides a proof-of-concept that the gut microbiota is informative in examining individualized responses to vitamin D supplementation, presenting a rationale for planning future clinical trials that focus on the inter and intra individual variation using multi-omics approaches such as genotyping, transcriptomics and proteomics.

## Methods

### Study participants and design

The study was approved by Qatar University (QU) Institutional Review Board (IRB) (QU-IRB; 531-A/15) and by Sidra Medicine IRB (1,705,010,938). The Investigators ensured that the study was conducted in full conformity with the current revision of the Declaration of Helsinki and with the ICH Guidelines for Good Clinical Practice (CPMP/ICH/135/95) July 1996. One hundred female students from QU were recruited for the study starting March 2018. Follow-up for the last subject was completed in September 2018. All subjects enrolled were healthy and did not have any underlying diseases or conditions. Subjects were excluded if they were taking vitamin D, antibiotics or were suffering from any chronic disease. Subjects were excluded from the final analysis if they failed to provide the blood or stool sample at either pre- or post- supplementation sampling points.

A total of 80 subjects were enrolled in the study after considering all the inclusion and exclusion criteria (Fig. 1A). Participants received the explanation about study aims and procedures before starting the intervention. All individuals were asked to complete a questionnaire that included present and past medical history, supplementation, dietary habits, exposure to sunlight and other details for the study. All participants underwent a physical examination and submitted their informed consent before inclusion. After the baseline assessment, blood and stool samples were collected and each participant received a weekly oral dose of 50,000 IU vitamin D_3_ (Nivagen pharmaceuticals, USA) to be taken for 12 weeks (phase I-baseline-pre-supplementation with vitamin D3). To encourage compliance, subjects were notified via phone messages to take their pills each week and were tested based on the pill count at the 12 weeks follow-up visit, where blood and stool samples were collected again (phase II- post-supplementation). Participants were asked to maintain their regular diet and eating practices. Intake of dairy products (milk, cheese, yogurt and butter/margarine) and fish was recorded for each participant as these are considered possible confounders of dietary vitamin D level.

At the end of the intervention, participants were classified as either responders to vitamin D supplementation (those who achieved serum levels of 25(OH) D above 20 ng/ml) or non-responders (those whose serum levels of 25(OH) D remained < 20 ng/ml)^37,38^.

### Sample collection and biochemical measures

Around 4 ml of peripheral blood was collected after overnight fasting from each participant in phase I (baseline-pre-supplementation) and in phase II (post-supplementation). Whole blood samples were centrifuged and separated within 3 h of venipuncture, and serum portions were frozen at − 80 °C for future measurement of creatinine, calcium, blood urea nitrogen (BUN)*,* aspartate aminotransferase (AST), alanine aminotransferase (ALT), and 25-hydroxyvitamin [25(OH)D] levels. ALT, AST, BUN, calcium and creatinine were measured using EasyRA analyzer and 25-hydroxyvitamin [25(OH)D] was measured using the DIAsource 25OH vitamin D Total ELISA 90′ Kit (catalog number: KAP1971/F1).

### Microbial DNA extraction from stool samples

A fraction of the collected stool sample (400–500 mg) was transferred to the OMNIgene GUT kit (DNA Genotek Inc, Ottawa, Canada), according to the manufacturer’s protocol. QIAamp Fast DNA Stool Mini Kit was used for fecal DNA extraction according to the manufacturer’s protocols. The DNA concentration and purity were evaluated using a Nanodrop spectrophotometer (Thermo Scientific, Wilmington, DE, USA). The extracted DNA samples were stored at − 20 °C until library preparation.

### DNA sequencing and gut microbial profiling

#### PCR amplification and high throughput sequencing

The 16S rRNA variable regions V3 and V4 were amplified with polymerase chain reaction (PCR), using the Illumina recommended amplicon primers:

Forward: 5′TCGTCGGCAGCGTCAGATGTGTATAAGAGACAGCCTACGGGNGGCWGCAG;

Reverse: 5 GTCTCGTGGGCTCGGAGATGTGTATAAGAGACAGGACTACHVGGGTATCTAATCC.

The PCR mixture comprised 5 μl of each forward and reverse primer (1 μM), 2.5 μl of template DNA for the samples, and 12.5 μl of 1× Hot Master Mix (Phusion Hot start Master Mix) to a final volume of 25 μl. The amplifications were performed under the following conditions: initial denaturation at 95 °C for 2 min, followed by 30 cycles of denaturation at 95 °C for 30 s, primer annealing at 60 °C for 30 s, and extension at 72 °C for 30 s, with a final elongation at 72 °C for 5 min. The presence of PCR products was visualized by electrophoresis using a 1.5% agarose gel. All amplicons were cleaned and sequenced according to the Illumina MiSeq 16S Metagenomic Sequencing Library Preparation protocol (http://support.illumina.com/downloads/16s_metagenomic_sequencing_library_preparation.html). Samples were multiplexed using a dual-index approach with the Nextera XT Index kit (Illumina, San Diego, USA) according to the manufacturer’s instructions. Amplicon library concentrations were determined using the Qubit HS dsDNA assay kit (Life Technologies, Australia). The final library was paired end sequenced at 2 × 300 bp using a MiSeq Reagent Kit v3 on Illumina MiSeq platform (Illumina, San Diego, USA), at the Sidra research facility.

#### 16S sequence data processing and statistical analysis

The sequencing quality was evaluated using Fast QC [http://www.bioinformatics.babraham.ac.uk/projects/fastqc] and the demultiplexed sequencing data imported into Quantitative Insights into Microbial Ecology (QIIME2; version 2019.4.0) software package^83,84^ [https://qiime2.org/]. Although the overall distribution was uniform across pre- and post- supplementation samples (Supplementary Fig. S10), several samples such as 33, 70 and 74 exhibited unequal distribution. The data were normalized to overcome the inherent bias in amplicon sequencing, as discussed below. The rarefaction curves tapered phylogenetically as the sequencing depth increased, implying that the entire microbial population was sufficiently represented (Supplementary Figs. S11 and S12) and the samples were rarefied at a depth of > 10,000. Samples from subjects 33, 70 and 74 were removed from the final analysis because of low sampling depth and the skewed distribution noted above. The data were denoised with DADA2^85^—this multiple step process runs from read filtering to dereplication to chimera removal. Both paired reads were trimmed from the forward end and read length of at least 250 bp for further processing to generate the amplicon sequence variant (ASV), or interchangeably called operational taxonomic units (OTUs). Taxonomic classification was performed utilizing 16S rRNA gene database from Greengenes (http://greengenes.lbl.gov)^86^ (version 13_8). The OTUs were classified using QIIME2 and the data imported into R (RStudio v 1.2 with R v 3.6)^87^ in a Biological Observation Matrix (biom) format, before further evaluation with the Phyloseq package^88^ among others. The final set of ASVs/OTUs was finally utilized for taxonomical classification using a pre-trained classifier (trained at 99% OTU full-length sequences) against Greengenes database 13_8 as provided by Qiime2^83,84^. For normalization, we utilized a random subsampling or the rarefaction on OTUs count. We also performed nonparametric statistical testing utilizing two-tailed Wilcoxon signed rank test for paired analysis^89^, and calculated the false discovery rate (FDR) with Bonferroni correction and resulting *p* value < 0.05 considered significant for all tests.

Alpha Diversity (within sample community) was assessed by observed OTUs (i.e., sum of unique OTUs per sample), Chao1^90^ (abundance based richness estimators, which is sensitive to rare OTUs), Shannon^91^ and inverse Simpson (InvSimpson)^92^ (which is more dependent on highly abundant OTUs and less sensitive to rare OTUs) indices in RStudio using the R package “vegan” (v2.5–6)^93^. Beta Diversity (Divergence in community composition between samples) was assessed using four different distance metrics: Weighted Unifrac, Unweighted Unifrac, Bray–Curtis (abundance) and Jaccard. PCA was used as an ordination method and significance was determined using the Adonis test (PERMANOVA) which considers the multidimensional structure of the data (e.g., compares entire microbial communities) to determine the significance (999 permutations). The B/F ratio was calculated with a mixed model for repeated measures controlling for random subject-specific effects with the LME4 package^94^.

Metagenome functional contents were analyzed using the PICRUSt software package (v1.0.0) to predict gene contents and metagenomic functional information^46^. The statistical evaluation was then performed with STAMP^95^ and significant pathways (*p* value < 0.05, CI 99%) were exported and used to generate the heatmap shown in (Supplementary Fig. S8).

To delineate the differentially abundant bacterial taxa in responders/non-responders to vitamin D supplementation we used DESeq2^96^. In the differential abundance analysis, rarefaction may lead to a lower power^97^; thus DESeq2 analysis was carried out on the un-rarefied data to allow maximum participation of sequenced reads (taking the entire data into consideration) using the DESeq2 inbuilt library size normalization facility.

### Ethics approval and consent to participate/publish

The study was approved by Qatar University (QU) Institutional Review Board (IRB) (QU-IRB; 531-A/15) and by Sidra Medicine IRB (1705010938). Informed consent to participate in and publish the study was obtained from all the participants and/or their legal guardians.

## Supplementary information

Supplementary information.

## Data Availability

The data is available upon request.

## References

[1] Hollander D, Truscott TC (1976). Mechanism and site of small intestinal uptake of vitamin D3 in pharmacological concentrations. Am. J. Clin. Nutr..

[2] Bell TD, Demay MB, Burnett-Bowie S-AM (2010). The biology and pathology of vitamin D control in bone. J. Cell. Biochem..

[3] Friedman PA, Gesek FA (1995). Cellular calcium transport in renal epithelia: Measurement, mechanisms, and regulation. Physiol. Rev..

[4] Aranow C (2011). Vitamin D and the immune system. J. Investig. Med..

[5] Forrest KYZ, Stuhldreher WL (2011). Prevalence and correlates of vitamin D deficiency in US adults. Nutr. Res..

[6] Cashman KD (2016). Vitamin D deficiency in Europe: Pandemic?. Am. J. Clin. Nutr..

[7] Sharif EA, Rizk NM (2011). The prevalence of vitamin D deficiency among female college students at Qatar University. Saudi Med. J..

[8] Al-Dabhani K (2017). Prevalence of vitamin D deficiency and association with metabolic syndrome in a Qatari population. Nutr. Diabetes.

[9] Singh P, Kumar M, Al Khodor S (2019). Vitamin D deficiency in the gulf cooperation council: Exploring the triad of genetic predisposition, the gut microbiome and the immune system. Front. Immunol..

[10] Biobank, Q. (Qatar, 2019).

[11] Manson JE (2019). Vitamin D supplements and prevention of cancer and cardiovascular disease. N. Engl. J. Med..

[12] Giovannucci E (2006). Prospective study of predictors of vitamin D status and cancer incidence and mortality in men. J. Natl. Cancer Inst..

[13] Dobnig H (2008). Independent association of low serum 25-hydroxyvitamin d and 1,25-dihydroxyvitamin d levels with all-cause and cardiovascular mortality. Arch. Intern. Med..

[14] Afzal S, Brondum-Jacobsen P, Bojesen SE, Nordestgaard BG (2014). Vitamin D concentration, obesity, and risk of diabetes: A mendelian randomisation study. Lancet Diabetes Endocrinol..

[15] Hypponen E, Laara E, Reunanen A, Jarvelin MR, Virtanen SM (2001). Intake of vitamin D and risk of type 1 diabetes: A birth-cohort study. Lancet.

[16] Nielsen OH, Rejnmark L, Moss AC (2018). Role of Vitamin D in the Natural History of Inflammatory Bowel Disease. J. Crohns Colitis.

[17] Garg M (2018). The effect of vitamin D on intestinal inflammation and faecal microbiota in patients with ulcerative colitis. J. Crohn's Colitis.

[18] Kampmann U (2014). Effects of 12 weeks high dose vitamin D3 treatment on insulin sensitivity, beta cell function, and metabolic markers in patients with type 2 diabetes and vitamin D insufficiency—a double-blind, randomized, placebo-controlled trial. Metabolism.

[19] Raftery T (2015). Effects of vitamin D supplementation on intestinal permeability, cathelicidin and disease markers in Crohn's disease: Results from a randomised double-blind placebo-controlled study. United Eur. Gastroenterol. J..

[20] Barengolts E (2013). Vitamin D and prebiotics may benefit the intestinal microbacteria and improve glucose homeostasis in prediabetes and type 2 diabetes. Endocr. Pract..

[21] Cantorna MT (2019). Vitamin D regulates the microbiota to control the numbers of RORgammat/FoxP3+ regulatory T cells in the colon. Front. Immunol..

[22] Luthold RV, Fernandes GR, Franco-de-Moraes AC, Folchetti LG, Ferreira SR (2017). Gut microbiota interactions with the immunomodulatory role of vitamin D in normal individuals. Metabolism.

[23] Kanhere M (2018). Bolus weekly vitamin D3 supplementation impacts gut and airway microbiota in adults with cystic fibrosis: A double-blind, randomized, placebo-controlled clinical trial. J. Clin. Endocrinol. Metab..

[24] Cantarel BL (2015). Gut microbiota in multiple sclerosis: Possible influence of immunomodulators. J. Investig. Med..

[25] Ciubotaru I, Green SJ, Kukreja S, Barengolts E (2015). Significant differences in fecal microbiota are associated with various stages of glucose tolerance in African American male veterans. Transl. Res..

[26] Waterhouse M (2019). Vitamin D and the gut microbiome: A systematic review of in vivo studies. Eur. J. Nutr..

[27] Naderpoor N (2019). Effect of vitamin D supplementation on faecal microbiota: A randomised clinical trial. Nutrients.

[28] Bashir M (2016). Effects of high doses of vitamin D3 on mucosa-associated gut microbiome vary between regions of the human gastrointestinal tract. Eur. J. Nutr..

[29] Charoenngam N, Shirvani A, Kalajian TA, Song A, Holick MF (2020). The effect of various doses of oral vitamin D3 supplementation on gut microbiota in healthy adults: A randomized, double-blinded dose-response study. Anticancer Res..

[30] Aloia JF (2008). Vitamin D intake to attain a desired serum 25-hydroxyvitamin D concentration. Am. J. Clin. Nutr..

[31] Gallagher JC, Sai A, Templin T, Smith L (2012). Dose response to vitamin D supplementation in postmenopausal women: A randomized trial. Ann. Intern. Med..

[32] Heaney RP, Davies KM, Chen TC, Holick MF, Barger-Lux MJ (2003). Human serum 25-hydroxycholecalciferol response to extended oral dosing with cholecalciferol. Am. J. Clin. Nutr..

[33] Talwar SA, Aloia JF, Pollack S, Yeh JK (2007). Dose response to vitamin D supplementation among postmenopausal African American women. Am. J. Clin. Nutr..

[34] Carlberg C, Haq A (2018). The concept of the personal vitamin D response index. J. Steroid Biochem. Mol. Biol..

[35] Zittermann A, Ernst JB, Gummert JF, Borgermann J (2014). Vitamin D supplementation, body weight and human serum 25-hydroxyvitamin D response: A systematic review. Eur. J. Nutr..

[36] Holick MF (2011). Evaluation, treatment, and prevention of vitamin D deficiency: An Endocrine Society clinical practice guideline. J. Clin. Endocrinol. Metab..

[37] 37Al-Daghri, N. M. *et al.* IGF and IGFBP as an index for discrimination between vitamin D supplementation responders and nonresponders in overweight Saudi subjects. *Medicine (Baltimore)* **97**, e0702, doi:10.1097/MD.0000000000010702 (2018). 10.1097/MD.0000000000010702 PMC5959419 29742726

[38] Al-Daghri NM (2019). Efficacy of vitamin D supplementation according to vitamin D-binding protein polymorphisms. Nutrition.

[39] Iruzubieta P, Teran A, Crespo J, Fabrega E (2014). Vitamin D deficiency in chronic liver disease. World J. Hepatol..

[40] Franca Gois PH, Wolley M, Ranganathan D, Seguro AC (2018). Vitamin D deficiency in chronic kidney disease: Recent evidence and controversies. Int. J. Environ. Res. Public Health.

[41] Bentli R (2013). Significant independent predictors of vitamin D deficiency in inpatients and outpatients of a nephrology unit. Int. J. Endocrinol..

[42] Bahreynian M (2018). Association of Serum 25-hydroxyvitamin D levels and liver enzymes in a nationally representative sample of Iranian adolescents: The childhood and adolescence surveillance and prevention of adult noncommunicable disease study. Int. J. Prev. Med..

[43] Arumugam M (2011). Enterotypes of the human gut microbiome. Nature.

[44] Eckburg PB (2005). Diversity of the human intestinal microbial flora. Science.

[45] Kuznetsova A, Brockhoff PB, Christensen RH (2017). lmerTest package: Tests in linear mixed effects models. J. Stat. Softw..

[46] Langille MG (2013). Predictive functional profiling of microbial communities using 16S rRNA marker gene sequences. Nat. Biotechnol..

[47] Rossi M, Amaretti A, Raimondi S (2011). Folate production by probiotic bacteria. Nutrients.

[48] D'Aimmo MR, Mattarelli P, Biavati B, Carlsson NG, Andlid T (2012). The potential of bifidobacteria as a source of natural folate. J. Appl. Microbiol..

[49] Guasch-Ferre M (2016). Metabolomics in prediabetes and diabetes: A systematic review and meta-analysis. Diabetes Care.

[50] Neis EP, Dejong CH, Rensen SS (2015). The role of microbial amino acid metabolism in host metabolism. Nutrients.

[51] Ley RE, Turnbaugh PJ, Klein S, Gordon JI (2006). Microbial ecology: Human gut microbes associated with obesity. Nature.

[52] Sanz Y, Moya-Perez A (2014). Microbiota, inflammation and obesity. Adv. Exp. Med. Biol..

[53] Mariat D (2009). The firmicutes/bacteroidetes ratio of the human microbiota changes with age. BMC Microbiol..

[54] Yang T (2015). Gut dysbiosis is linked to hypertension. Hypertension.

[55] Everard A (2011). Responses of gut microbiota and glucose and lipid metabolism to prebiotics in genetic obese and diet-induced leptin-resistant mice. Diabetes.

[56] Miller RS, Hoskins LC (1981). Mucin degradation in human colon ecosystems. Fecal population densities of mucin-degrading bacteria estimated by a "most probable number" method. Gastroenterology.

[57] Derrien M (2010). Mucin-bacterial interactions in the human oral cavity and digestive tract. Gut Microbes.

[58] Derrien M, Collado MC, Ben-Amor K, Salminen S, de Vos WM (2008). The Mucin degrader Akkermansia muciniphila is an abundant resident of the human intestinal tract. Appl. Environ. Microbiol..

[59] Everard A (2013). Cross-talk between Akkermansia muciniphila and intestinal epithelium controls diet-induced obesity. Proc. Natl. Acad. Sci. USA.

[60] Shin NR (2014). An increase in the Akkermansia spp. population induced by metformin treatment improves glucose homeostasis in diet-induced obese mice. Gut.

[61] Hansen CH (2014). A maternal gluten-free diet reduces inflammation and diabetes incidence in the offspring of NOD mice. Diabetes.

[62] Roopchand DE (2015). Dietary polyphenols promote growth of the gut bacterium akkermansia muciniphila and attenuate high-fat diet-induced metabolic syndrome. Diabetes.

[63] Hansen CH (2012). Early life treatment with vancomycin propagates Akkermansia muciniphila and reduces diabetes incidence in the NOD mouse. Diabetologia.

[64] Ruiz L, Delgado S, Ruas-Madiedo P, Sanchez B, Margolles A (2017). Bifidobacteria and their molecular communication with the immune system. Front. Microbiol..

[65] Pompei A (2007). Administration of folate-producing bifidobacteria enhances folate status in Wistar rats. J. Nutr..

[66] Wu GD (2011). Linking long-term dietary patterns with gut microbial enterotypes. Science (New York, N.Y.).

[67] Rigsbee L (2012). Quantitative profiling of gut microbiota of children with diarrhea-predominant irritable bowel syndrome. Am. J. Gastroenterol..

[68] Scher JU (2013). Expansion of intestinal Prevotella copri correlates with enhanced susceptibility to arthritis. eLife.

[69] Wu S (2010). Vitamin D receptor negatively regulates bacterial-stimulated NF-kappaB activity in intestine. Am. J. Pathol..

[70] Sun J (2010). Vitamin D and mucosal immune function. Curr. Opin. Gastroenterol..

[71] Wu S (2015). Intestinal epithelial vitamin D receptor deletion leads to defective autophagy in colitis. Gut.

[72] Jin D (2015). Lack of vitamin D receptor causes dysbiosis and changes the functions of the murine intestinal microbiome. Clin. Ther..

[73] Du J (2015). 1,25-Dihydroxyvitamin D protects intestinal epithelial barrier by regulating the myosin light chain kinase signaling pathway. Inflamm. Bowel Dis..

[74] Zhang YG (2015). Tight junction CLDN2 gene is a direct target of the vitamin D receptor. Sci. Rep..

[75] Golan MA (2015). Transgenic expression of vitamin D receptor in gut epithelial cells ameliorates spontaneous colitis caused by interleukin-10 deficiency. Dig. Dis. Sci..

[76] Appleyard CB (2011). Pretreatment with the probiotic VSL# 3 delays transition from inflammation to dysplasia in a rat model of colitis-associated cancer. Am. J. Physiol. Gastrointest. Liver Physiol..

[77] Waterhouse JC, Perez TH, Albert PJ (2009). Reversing bacteria-induced vitamin D receptor dysfunction is key to autoimmune disease. Ann. N. Y. Acad. Sci..

[78] Fangmann D (2018). Targeted microbiome intervention by microencapsulated delayed-release niacin beneficially affects insulin sensitivity in humans. Diabetes Care.

[79] Iqbal J, Hussain MM (2009). Intestinal lipid absorption. Am. J. Physiol. Endocrinol. Metab..

[80] Thompson GR (1989). Lipid related consequences of intestinal malabsorption. Gut.

[81] Maurya VK, Aggarwal M (2017). Factors influencing the absorption of vitamin D in GIT: An overview. J. Food Sci. Technol..

[82] Yang JY (2017). Gut commensal Bacteroides acidifaciens prevents obesity and improves insulin sensitivity in mice. Mucosal Immunol..

[83] Bolyen E (2019). Reproducible, interactive, scalable and extensible microbiome data science using QIIME 2. Nat. Biotechnol..

[84] Caporaso JG (2010). QIIME allows analysis of high-throughput community sequencing data. Nat. Methods.

[85] Callahan BJ (2016). DADA2: High-resolution sample inference from Illumina amplicon data. Nat. Methods.

[86] DeSantis TZ (2006). Greengenes, a chimera-checked 16S rRNA gene database and workbench compatible with ARB. Appl. Environ. Microbiol..

[87] R Core Team, R. (R foundation for statistical computing Vienna, Austria, 2013).

[88] McMurdie PJ, Holmes S (2013). phyloseq: An R package for reproducible interactive analysis and graphics of microbiome census data. PLoS ONE.

[89] Weiss S (2017). Normalization and microbial differential abundance strategies depend upon data characteristics. Microbiome.

[90] Chao A (1987). Estimating the population size for capture-recapture data with unequal catchability. Biometrics.

[91] Shannon CE (1948). A mathematical theory of communication, Part II. Bell Syst. Tech. J..

[92] Simpson EH (1949). Measurement of diversity. Nature.

[93] Jari Oksanen, F. G. B., Michael Friendly, Roeland Kindt, Pierre Legendre, D. M., Peter R. Minchin, R. B. O'Hara,, Gavin L. Simpson, P. S., M. Henry H. Stevens, Eduard Szoecs, & Wagner, H. (2019).

[94] Bates, D., Mächler, M., Bolker, B. & Walker, S. Fitting Linear Mixed-Effects Models Using lme4. **67**, 48. 10.18637/jss.v067.i01 (2015).

[95] Parks DH, Tyson GW, Hugenholtz P, Beiko RG (2014). STAMP: Statistical analysis of taxonomic and functional profiles. Bioinformatics.

[96] Love MI, Huber W, Anders S (2014). Moderated estimation of fold change and dispersion for RNA-seq data with DESeq2. Genome Biol..

[97] Kashani A (2019). Impaired glucose metabolism and altered gut microbiome despite calorie restriction of ob/ob mice. Anim. Microbiome.

