# Supplementary material for: The potential role of vitamin D supplementation as a gut microbiota modifier in healthy individuals
Source: Sci Rep. 2020 Dec 10;10:21641. doi: 10.1038/s41598-020-77806-4 (PMC7729960; doi:10.1038/s41598-020-77806-4)
Supplement: Supplementary file 1 — Supplementary information. [file 41598_2020_77806_MOESM1_ESM.pdf]

The potential role of vitamin D supplementation as a gut microbiota modifier in healthy individuals

Parul Singh[a], Arun Rawat[a], Mariam Alwakeel[b], Elham Sharif[b]*[#] and Souhaila Al Khodor[a]*[#]

[a]*Research Department, Sidra Medicine, Doha, Qatar,* [b]*College of Health Sciences, Qatar University Doha, Qatar*

[#] Equal senior authors

*Address correspondence to Dr. Souhaila Al Khodor: and Dr. Elham Sharif: e.sharif@qu.edu.qa

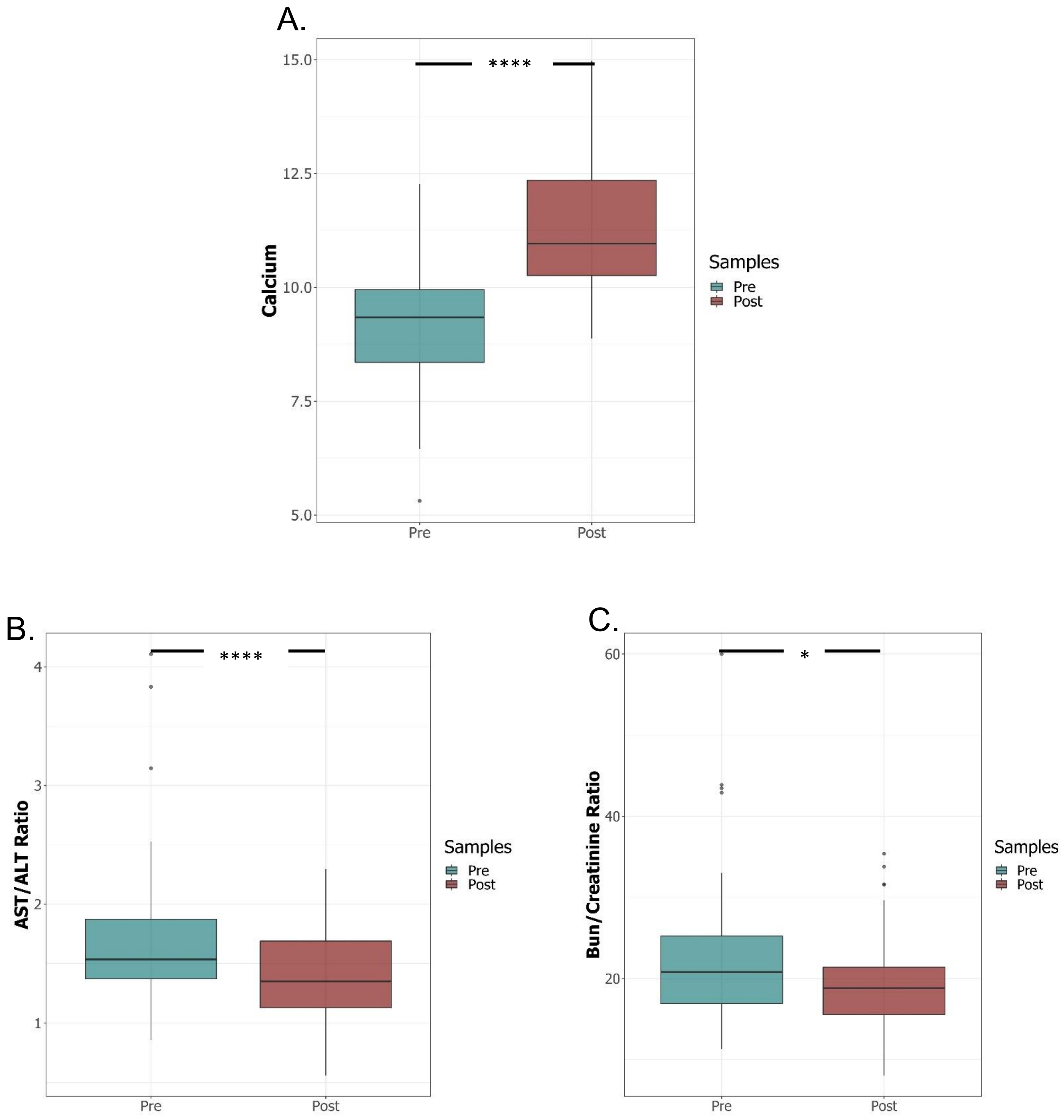

Supplementary Fig. S1 Blood samples were taken before pre- and post- 12 weeks of supplementation with vitamin D and assayed for levels of (**A**) Calcium(mg/dl)(Paired Wilcoxon; **** $p<0.0001$). Levels of (**B**) BUN(mg/dl) and creatinine(mg/dl), and (**C**) AST(U/L) and ALT(U/L) were also measured and are presented as ratios (Paired Wilcoxon;**** $p<0.0001$ and *$p=0.034$). The figure was generated using (RStudio v 1.2 with R v 3.6)[1]

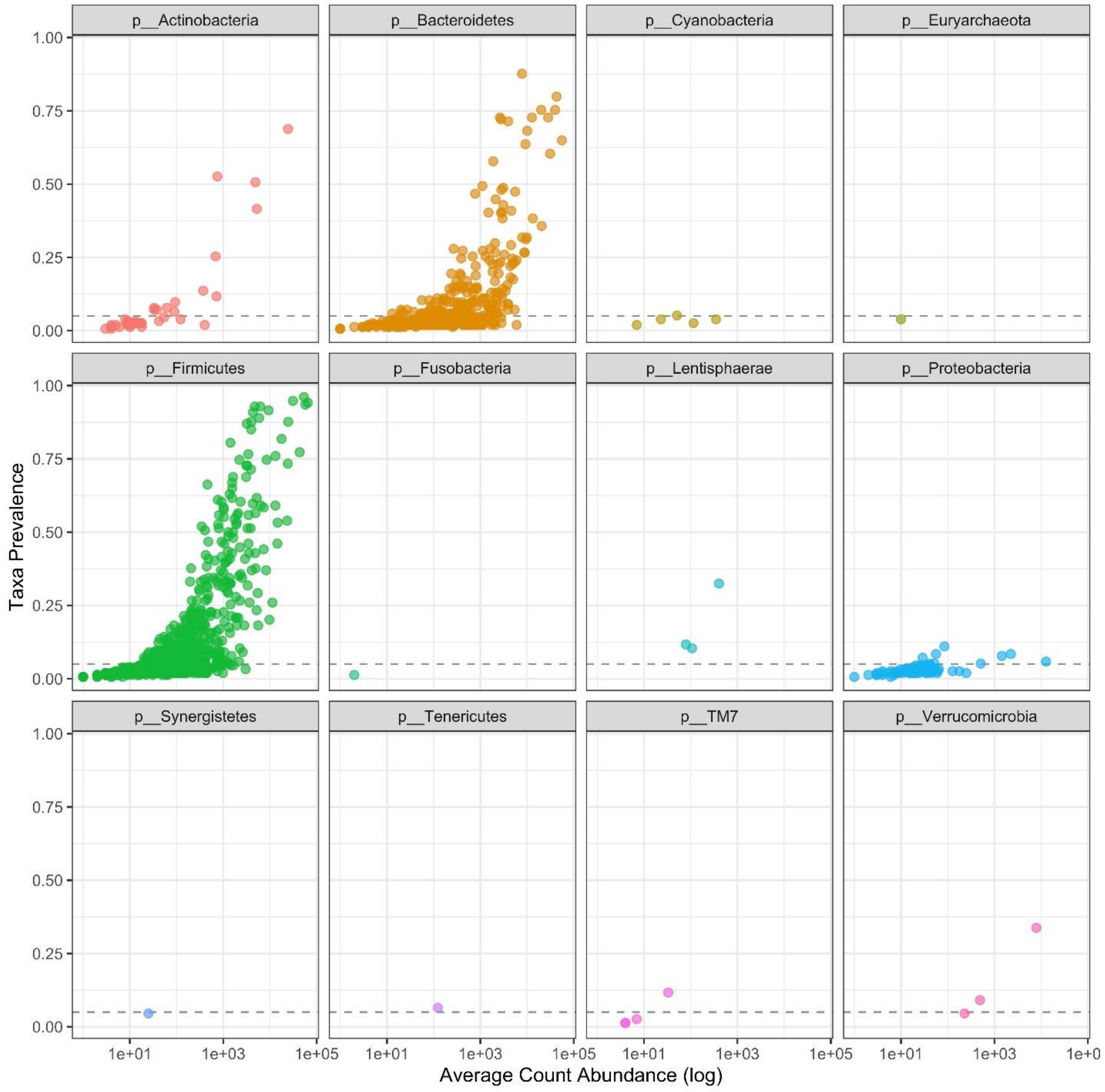

Supplementary Fig. S2 Prevalence plots (taxa prevalence versus average count) for bacterial taxa representing the phylum-level diversity across pre- and post- vitamin D supplementation stool samples from all women. Each point corresponds to a single taxon. Taxa prevalence is defined as the fraction of samples containing the taxa. The figure was generated using (RStudio v 1.2 with R v 3.6)[1]

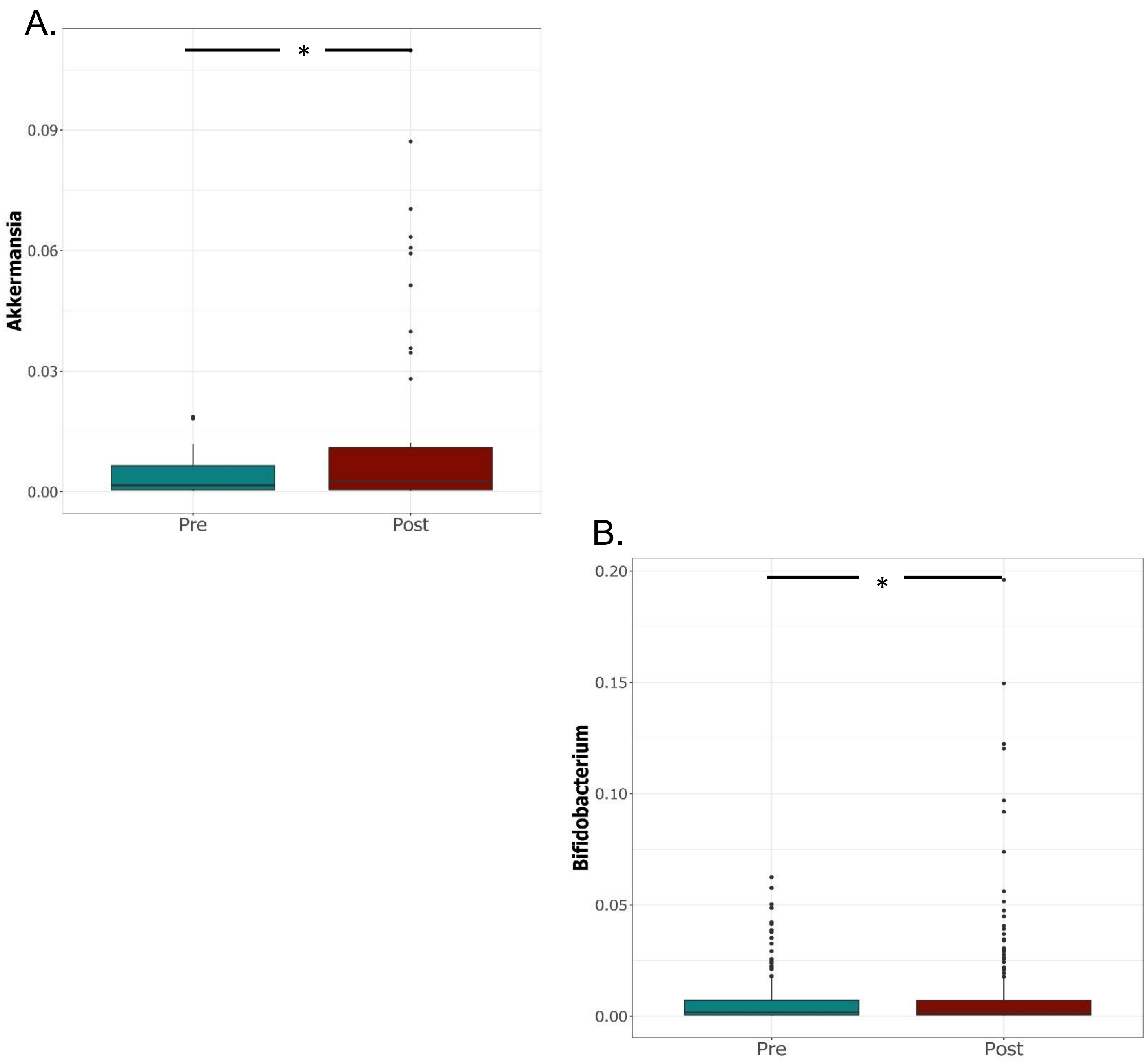

Supplementary Fig. S3 Box plot representation of the relative abundance of genera (**A**) *Akkermansia* and (**B**) *Bifidobacterium* (paired Wilcoxon; $* \, p < 0.05$). The figure was generated using (RStudio v 1.2 with R v 3.6)[1]

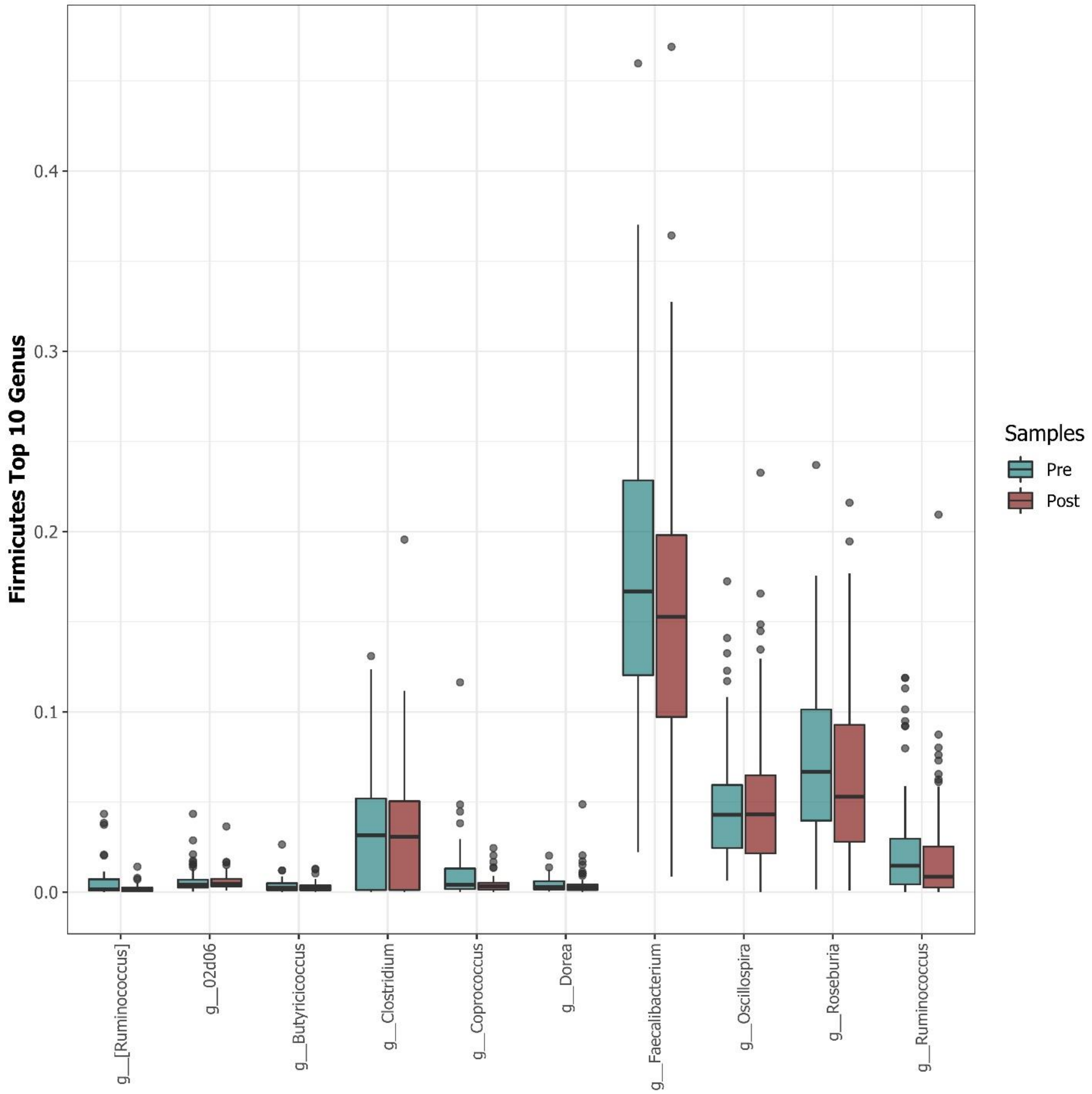

Supplementary Fig. S4 Box plot representation of the relative abundance of the top 10 genera in the phylum *Firmicutes*. The figure was generated using (RStudio v 1.2 with R v 3.6)[1]

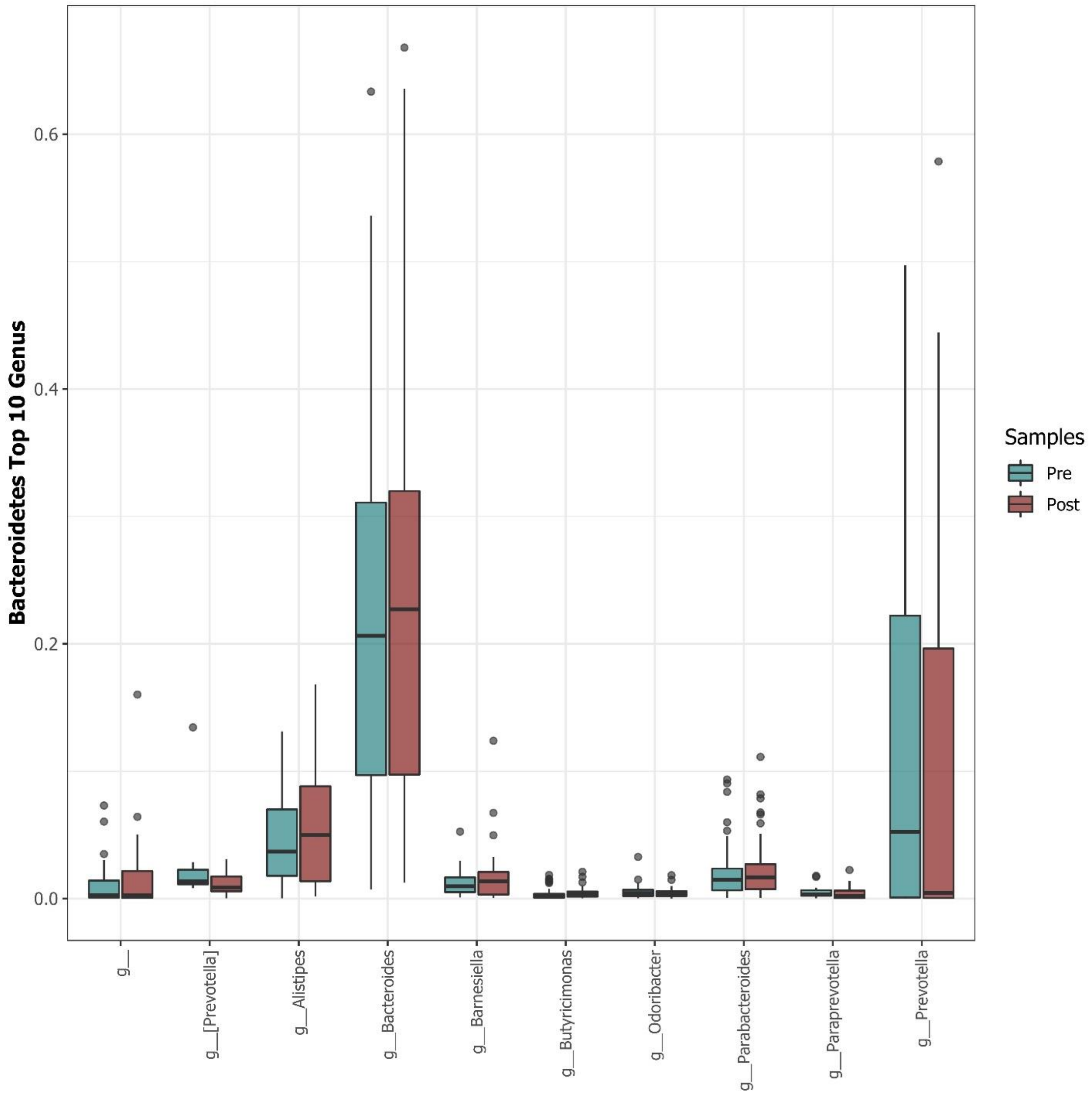

Supplementary Fig. S5 Box plot representation of the relative abundance of the top 10 genera in phylum Bacteroidetes. The figure was generated using (RStudio v 1.2 with R v 3.6)[1]

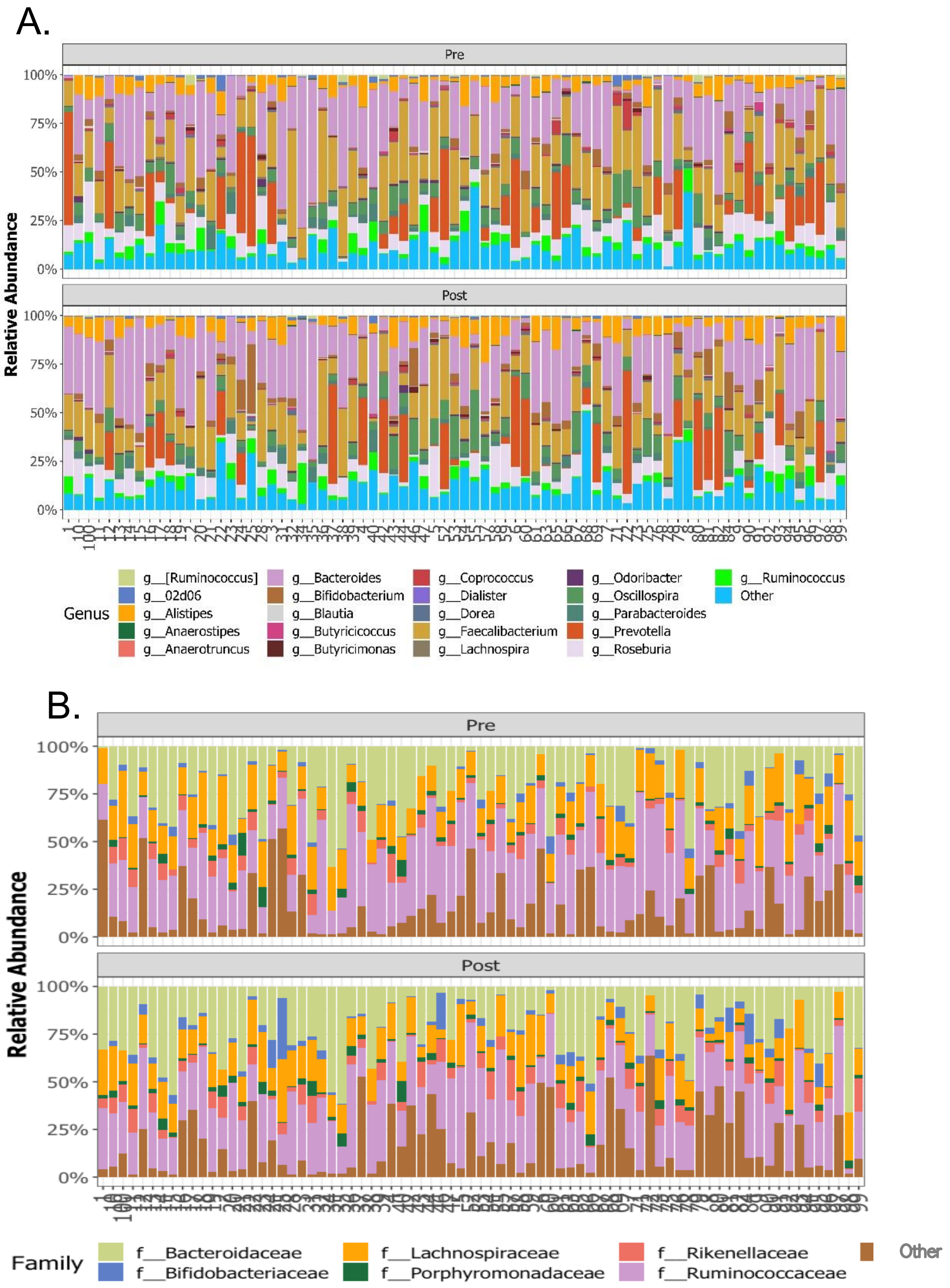

Supplementary Fig. S6 Box plot representation of the relative abundance at A. Genera and B. Family level per sample pre and post supplementation. The figure was generated using (RStudio v 1.2 with R v 3.6)[1]

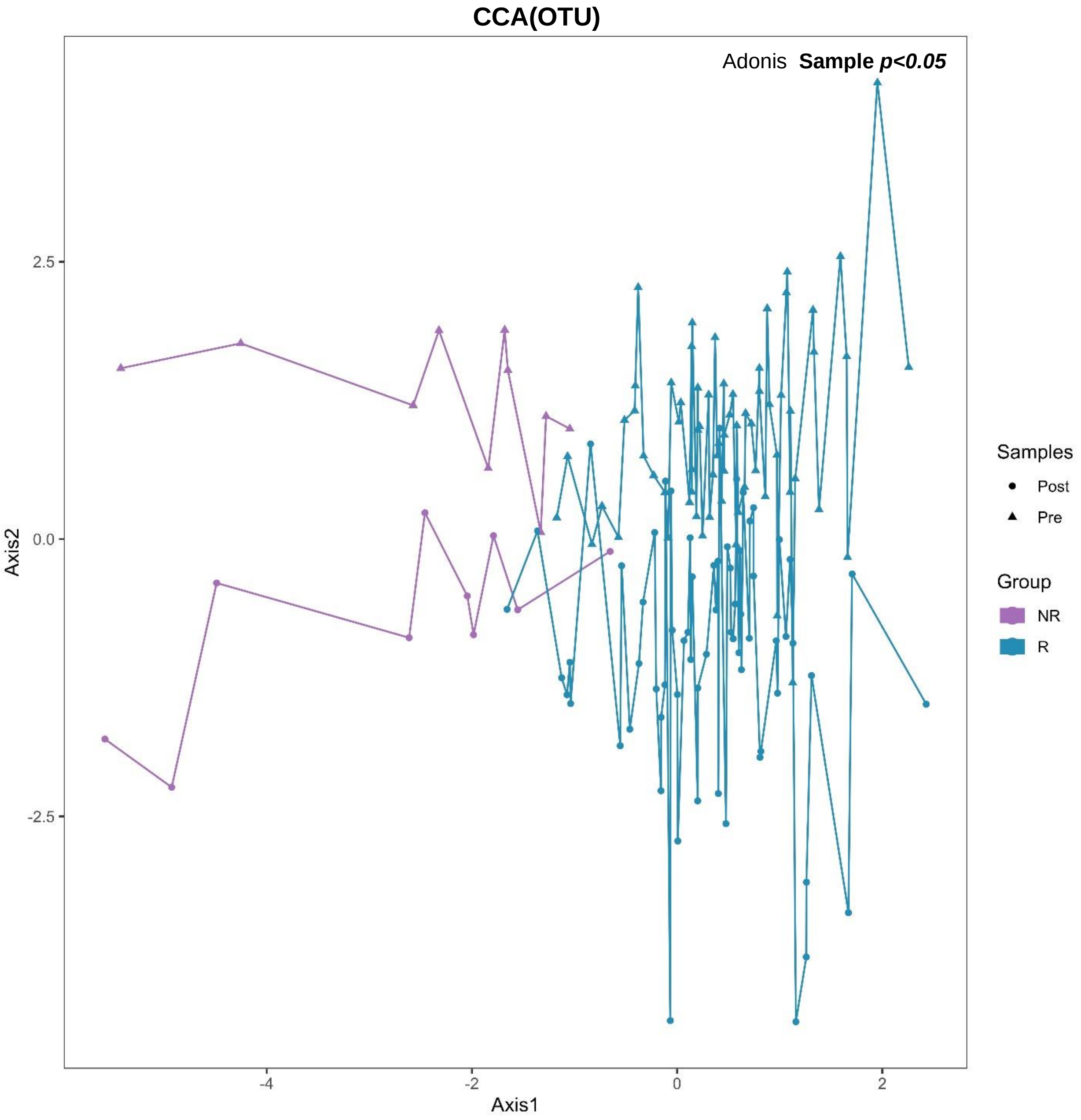

Supplementary Fig. S7 Canonical Correspondence Analysis (CCA) visualizing the compositional distribution of the fecal microbiota pre- and post- vitamin D supplementation in responders and non-responders. The figure was generated using (RStudio v 1.2 with R v 3.6)[1]

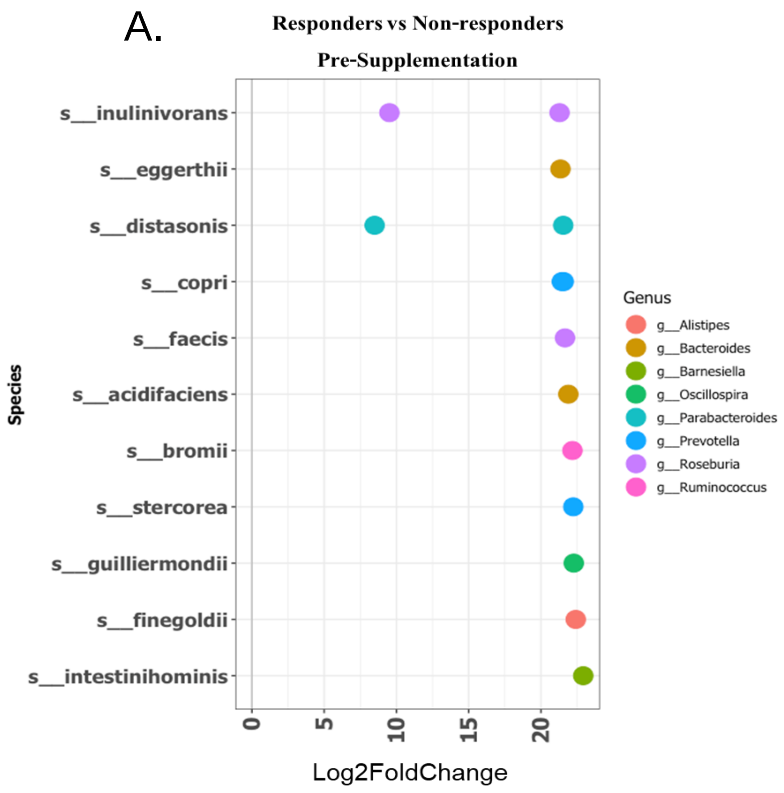

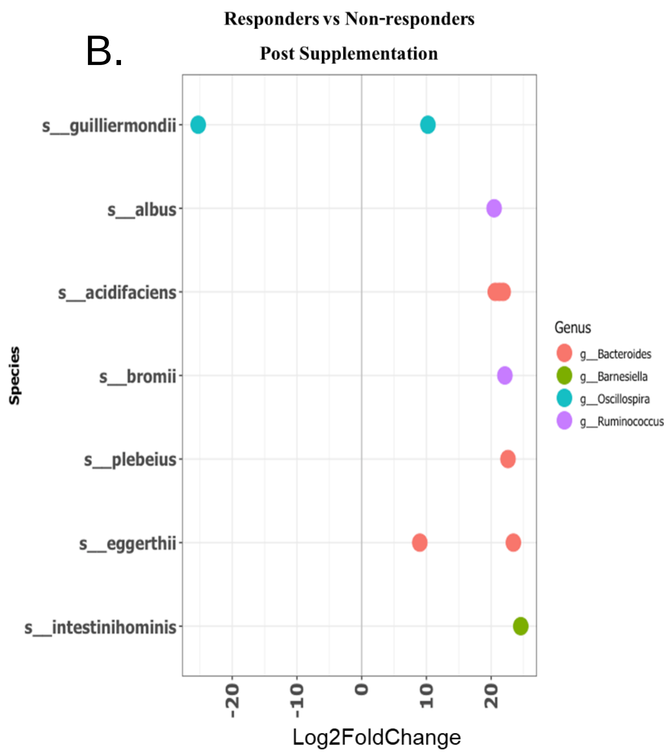

Supplementary Fig. S8 DESeq2 differential abundance analysis of significantly different OTUs responder's vs non-responder's (*p* adj< 0.05, FDR-corrected); A. Pre-supplementation B. Post-supplementation. OTUs to the right of the zero line were more abundant and OTUs to the left of the zero line were less abundant in responders compared to non-responders.

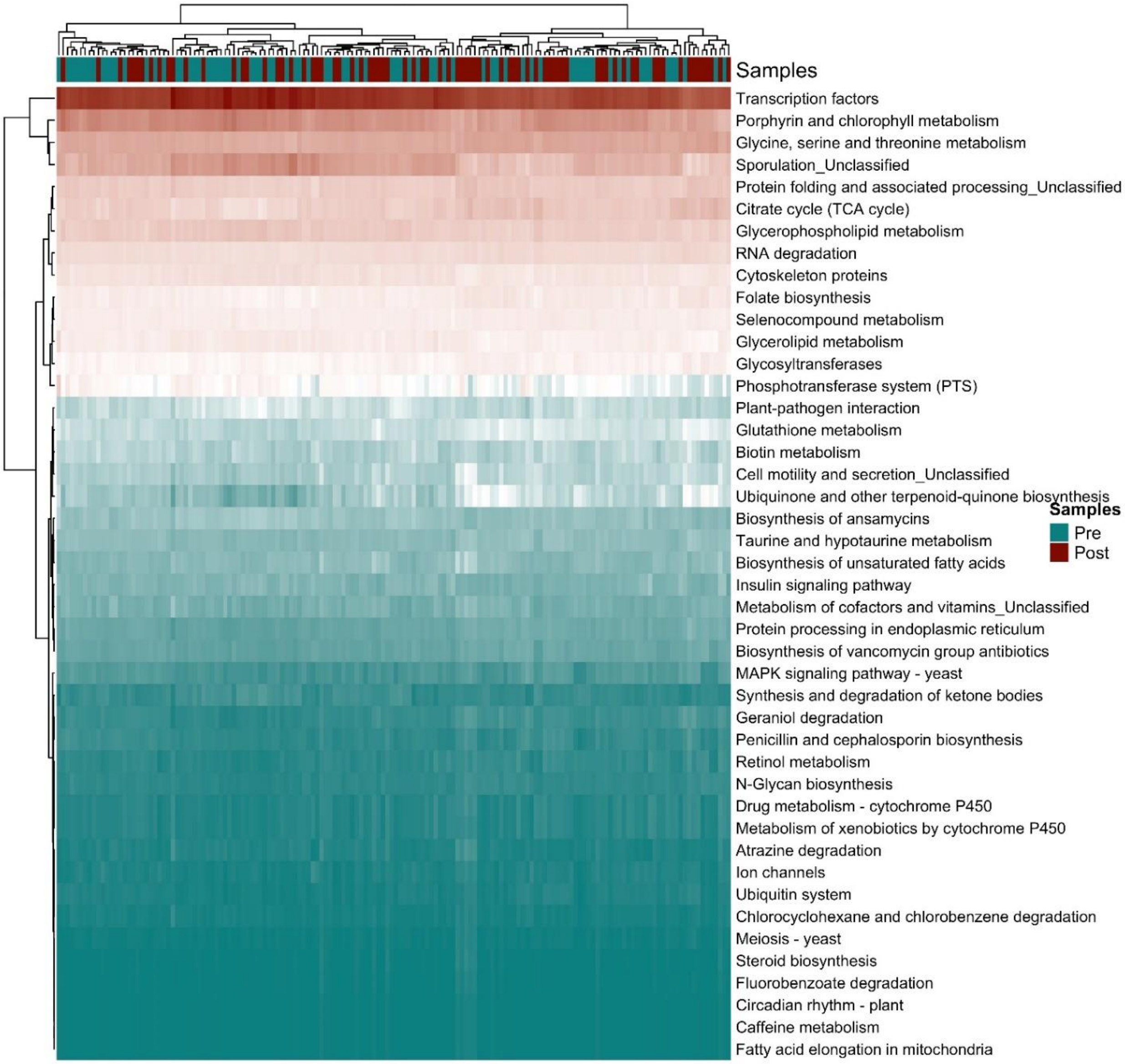

Supplementary Fig. S9 Heat map representation of predicted functional gene expression pathways with significantly altered expression between pre- and post- Vitamin D supplementation samples, identified using PICRUSt analysis of 16S rRNA phylogenetic sequencing data. The figure was generated using (RStudio v 1.2 with R v 3.6)[1]

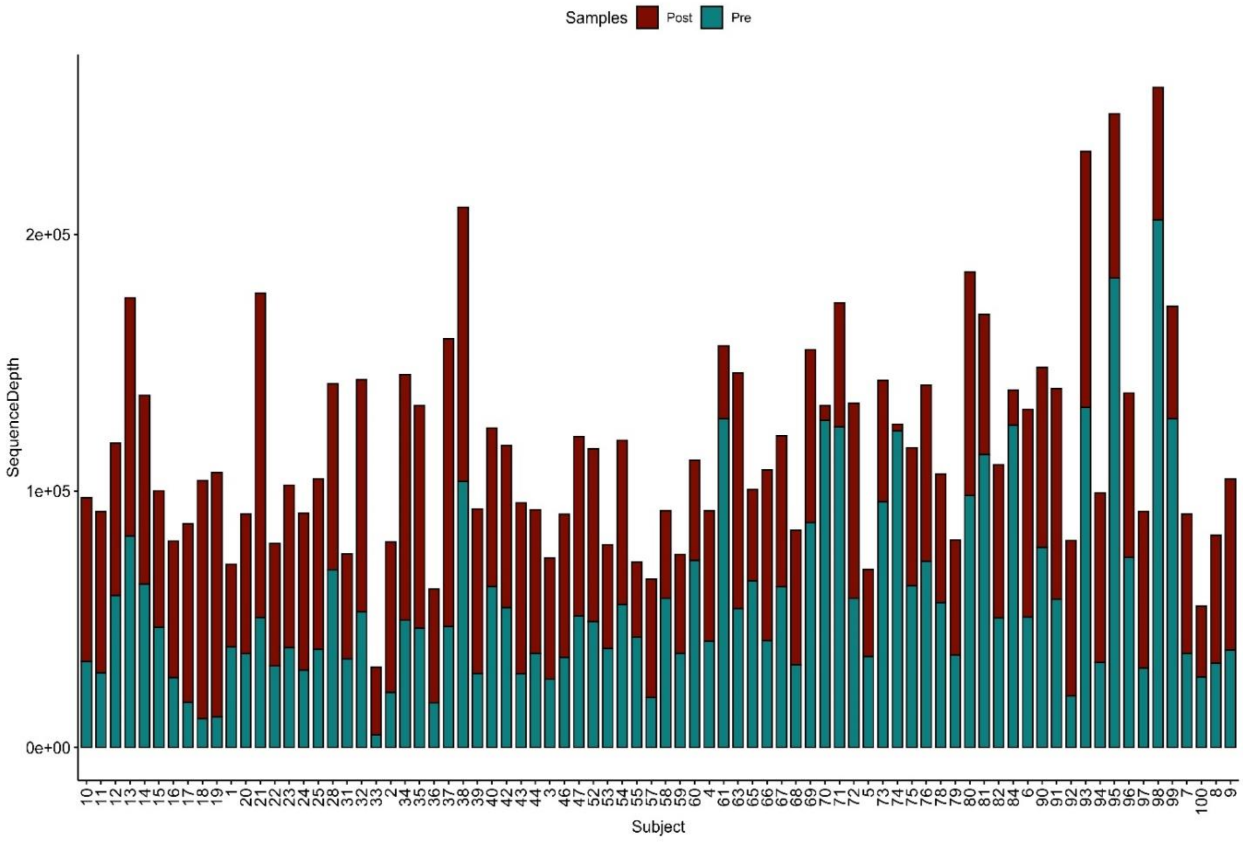

Supplementary Fig. S10 The figure depicts uniform coverage across the samples and adequate sequencing representation in each of the pre- and post- vitamin D supplementation stool samples. This is further validated with the rarefaction curve in Supplementary Fig. S11. The figure was generated using (RStudio v 1.2 with R v 3.6)[1]

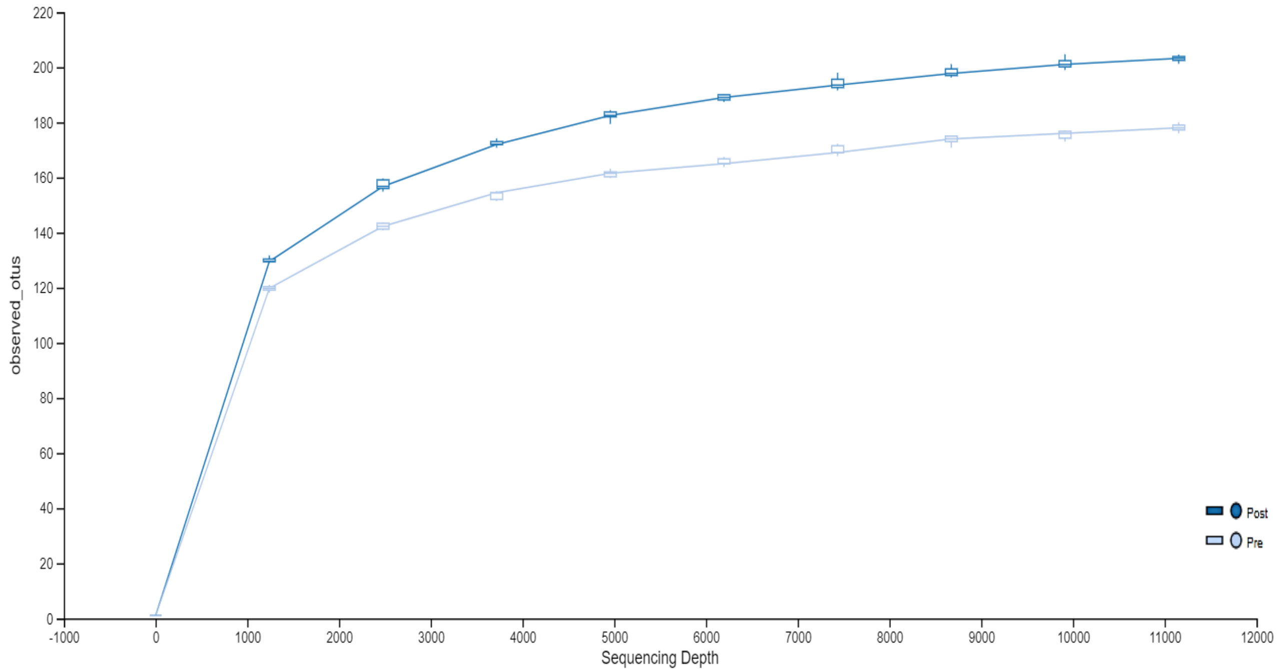

Supplementary Fig. S11 Rarefaction curves of 16S rRNA gene sequence representing species diversity between the pre- and post- vitamin D supplementation samples based on observed OTUs. X -axis reports the number of sequences per sample. The figure was generated using QIIME2 (Version 2018.11)[2]

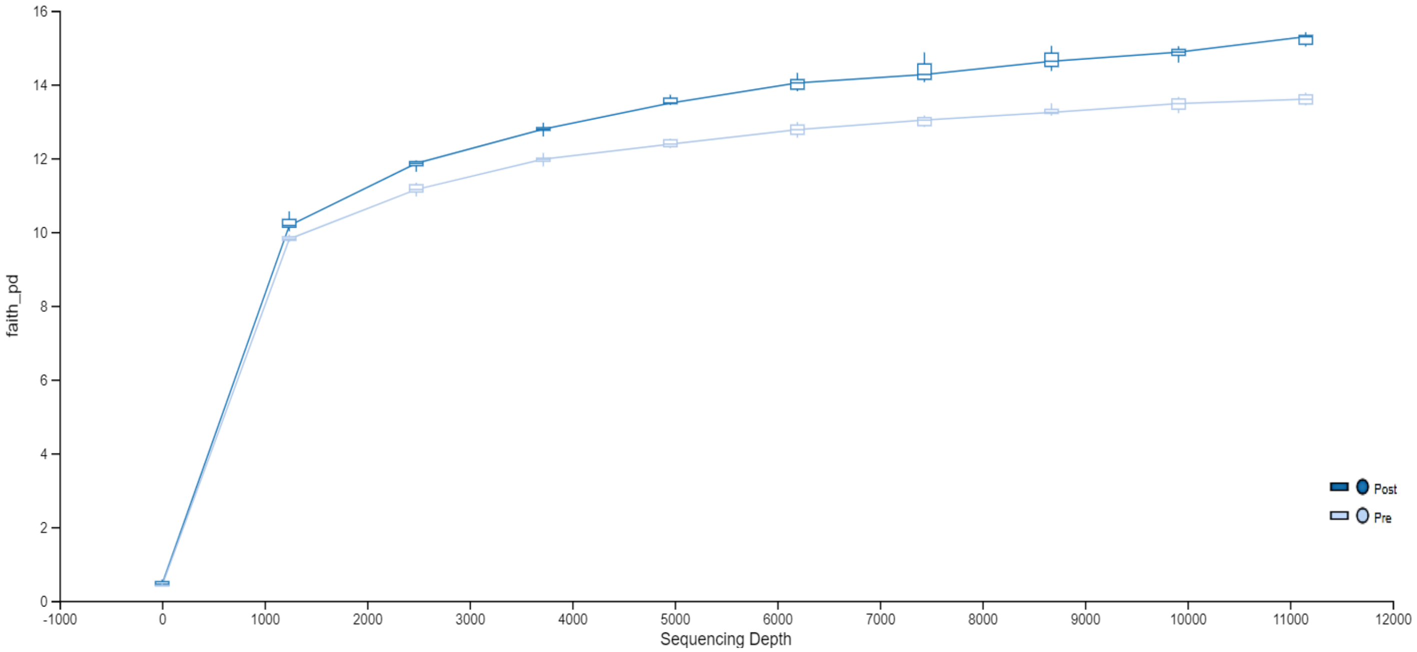

Supplementary Fig. S12 Rarefaction curves of 16S rRNA gene sequence representing species diversity between the pre- and post- vitamin D supplementation samples based on Faith-PD. X - axis reports the number of sequences per sample. The figure was generated using QIIME2 (Version 2018.11)[2]

1 R Core Team, R. (R foundation for statistical computing Vienna, Austria, 2013).
2 Caporaso, J. G. *et al.* QIIME allows analysis of high-throughput community sequencing data. *Nat Methods* **7**, 335-336, doi:10.1038/nmeth.f.303 (2010).
